# Deep-PK: deep learning for small molecule pharmacokinetic and toxicity prediction

**DOI:** 10.1093/nar/gkae254

**Published:** 2024-04-18

**Authors:** Yoochan Myung, Alex G C de Sá, David B Ascher

**Affiliations:** School of Chemistry and Molecular Biosciences, The Australian Centre for Ecogenomics, The University of Queensland, Brisbane, Queensland 4072, Australia; Computational Biology and Clinical Informatics, Baker Heart and Diabetes Institute, Melbourne, Victoria 3004, Australia; School of Chemistry and Molecular Biosciences, The Australian Centre for Ecogenomics, The University of Queensland, Brisbane, Queensland 4072, Australia; Computational Biology and Clinical Informatics, Baker Heart and Diabetes Institute, Melbourne, Victoria 3004, Australia; Baker Department of Cardiometabolic Health, The University of Melbourne, Parkville, Victoria 3010, Australia; School of Chemistry and Molecular Biosciences, The Australian Centre for Ecogenomics, The University of Queensland, Brisbane, Queensland 4072, Australia; Computational Biology and Clinical Informatics, Baker Heart and Diabetes Institute, Melbourne, Victoria 3004, Australia; Baker Department of Cardiometabolic Health, The University of Melbourne, Parkville, Victoria 3010, Australia

## Abstract

Evaluating pharmacokinetic properties of small molecules is considered a key feature in most drug development and high-throughput screening processes. Generally, pharmacokinetics, which represent the fate of drugs in the human body, are described from four perspectives: absorption, distribution, metabolism and excretion—all of which are closely related to a fifth perspective, toxicity (ADMET). Since obtaining ADMET data from *in vitro*, *in vivo* or pre-clinical stages is time consuming and expensive, many efforts have been made to predict ADMET properties via computational approaches. However, the majority of available methods are limited in their ability to provide pharmacokinetics and toxicity for diverse targets, ensure good overall accuracy, and offer ease of use, interpretability and extensibility for further optimizations. Here, we introduce Deep-PK, a deep learning-based pharmacokinetic and toxicity prediction, analysis and optimization platform. We applied graph neural networks and graph-based signatures as a graph-level feature to yield the best predictive performance across 73 endpoints, including 64 ADMET and 9 general properties. With these powerful models, Deep-PK supports molecular optimization and interpretation, aiding users in optimizing and understanding pharmacokinetics and toxicity for given input molecules. The Deep-PK is freely available at https://biosig.lab.uq.edu.au/deeppk/.

## Graphical Abstract

**Graphical Abstract:**
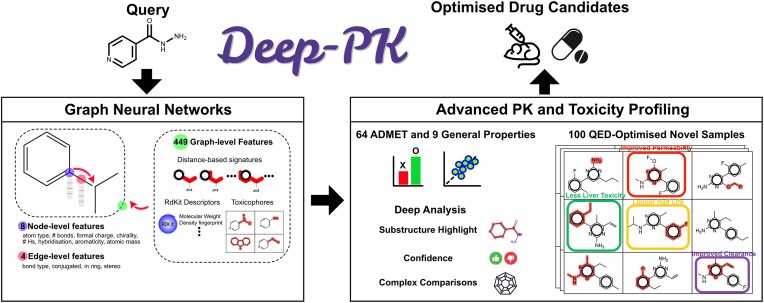

## Introduction

Drug discovery is a costly, lengthy and uncertain problem (1–7). Usually, the process of proposing a novel drug, from pre-clinical testing to approval, takes 12–15 years (5,8), on average, and the cost exceeds $2.5 billion (5,9,10). Most of the mapped hardness of this process emerges from the stringent multiple phases defined by regulatory agencies, which need to certify both the safety and potency of the new compounds during pre-clinical and clinical trials before their respective approval (11,12). In these phases, an inherent issue in drug discovery is the high attrition rate, where 80–90% of projects are discontinued before even getting tested in humans (5), and almost 95% of the drugs entering human trials fail (4,5,13). Along the same lines (14), estimates suggest that only 1 out of 5000–10 000 discovered compounds reaches the U.S. Food and Drug Administration (FDA)’s review phase (15).

Evaluating, understanding and optimizing pharmacokinetic and toxicity properties under the umbrella of ADMET (absorption, distribution, metabolism, elimination and toxicity) categories have become essential for reducing attrition rates in later phases of drug research and development (3,16–21). Over 80% of newly proposed drugs fail during development stages because of inadequate ADMET properties (22). It is estimated that success rates could increase significantly if ADMET properties were properly set at pre-clinical and clinical stages (23–26).

Both *in vivo* and *in vitro* assessments of small molecules have become important strategies for the initial selection or identification of chemical leads (27). Although being precise and useful in screening small molecules, reducing the overall number of molecules likely to present issues on pharmacokinetic and toxicity properties, *in vivo* and *in vitro* ADMET studies are expensive, inefficient and time consuming (in terms of human resources, equipment and laboratory operations) to assess the ADMET properties on the ever-growing number of developed compounds (25,28–30), besides the efforts on automation to tackle the drug screening bottleneck.

Under these circumstances, computational (*in silico*) approaches are a great alternative for pre-screening the high number of compounds being proposed on a day-to-day basis (25,30–35). Furthermore, *in vitro* or *in vivo* analyses are only possible after the molecule has already been synthesized (36), not being feasible to test them *a priori*. Hence, *in silico* ADMET evaluation is advantageous in terms of meeting the high compound throughput, allowing prioritization of compound libraries.

Several methods and tools have been proposed to promote ADMET evaluation, including SwissADME (34), ADMETlab (25,30), pkCSM (32), Interpretable-ADMET (35) and toxCSM (33). In spite of the computational effort, all these tools have major issues with integrating other important tasks apart from the predictive analysis, such as optimization and predictive molecular interpretation. In addition, the majority of the currently proposed tools do not consider a proper set of ADMET endpoints nor open their used experimental data. Unavailability and lack of scalability are also common drawbacks of current ADMET tools and methods. In summary, they are not able to accurately and correctly guide drug development research at scale.

We introduce Deep-PK, an advanced web-based tool designed to address the challenges and issues associated with small molecule pharmacokinetics and toxicity. Deep-PK stands out for its interpretability, accuracy and robustness, offering a comprehensive solution for the prediction, optimization, analysis and interpretation of these crucial profiles. It depends on the robustness of graph neural networks (GNNs) to build a set of 73 robust ADMET models. With predictive, optimization and interpretation features, Deep-PK yields a trustworthy platform for the development of safer, less toxic and highly bioavailable drugs.

## Materials and methods

### Data preparation

*Data collection and curation*. In Deep-PK, small molecule datasets on 73 endpoints were employed to train, (cross-)validate and test the deep learning models, including 49 and 24 that represent binary classification and regression tasks, respectively. These datasets correspond to 8 absorption, 5 distribution, 13 metabolism, 3 excretion and 35 toxicity experimental assays. In addition, 9 datasets involving general and complementary physicochemical properties were also used to assist in the characterization of small molecules (Supplementary Table S1). We collected the experimental ADMET data from four pharmacokinetic prediction methods, namely ADMETlab 2.0 (25), Interpretable-ADMET (35), toxCSM (33) and pkCSM (32).

**Figure 1.**
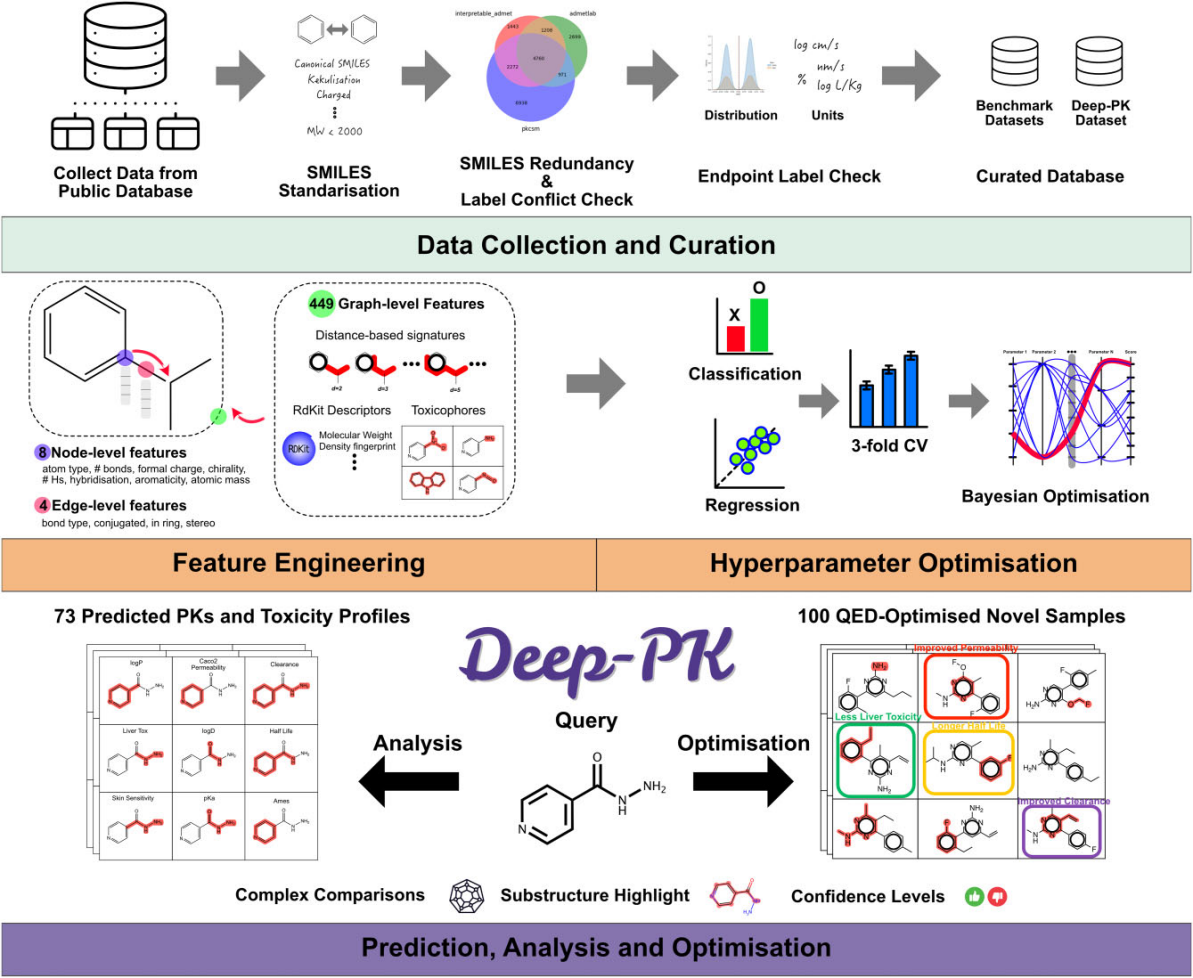
Deep-PK pipeline. In the data collection and curation steps, publicly available datasets were gathered and curated to eliminate redundancy, resolve label conflicts and ensure consistency in endpoint label units. These curated datasets were then utilized for preparing benchmark and Deep-PK datasets (Supplementary Table S1). In addition to the default node/edge features provided by Chemprop, our approach incorporated 449 graph-level features in the D-MPNN (directed message passing neural network) learning process. Hyperparameter optimization was carried out using Bayesian optimization with 3-fold cross-validation. Deep-PK provides predictions for 73 pharmacokinetic and toxicity endpoints, along with detailed information on corresponding molecules accessible through the analysis page. Users can explore optimization strategies for a query molecule through the optimization step, which includes 100 derivatives and predictions of ADMET and general properties.

Given the high level of heterogeneity among the small molecules and their respective ADMET targets, we proceeded with preprocessing steps to guarantee the validity and quality of the data. As shown in Figure 1, the first step involved converting all SMILES (simplified molecular-input line-entry system), which represent the small molecules, in their canonical form. Molecular sanitization using RDKit version 2022.9.3 (37) was also performed and included standardizing up to seven non-standard valence states, kekulizing aromatic rings, removing molecular weights ≥2000 and adding explicit hydrogens. Any molecules that failed at the canonization and sanitization steps were discarded from our acquired data.

The experimental labels were rigorously verified in the preprocessing pipeline of Deep-PK. SMILES with discrepancies such as different categorical labels, units or a variation of 20% in label values (for regression tasks) were identified and discarded from the dataset utilized to acquire ADMET experimental data for Deep-PK. The details of curated datasets are shown in Supplementary Table S1.

*Training, validation and test datasets*. We conducted a comprehensive assessment of the Deep-PK pipeline’s robustness and scalability through two distinct approaches. Initially, we utilized datasets from other tools, incorporating their training, validation and test split information. Subsequently, we aggregated all data points for each endpoint from various databases, creating what we refer to as the Deep-PK dataset.

To evaluate our deep learning pipeline, we specifically focused on the endpoints provided by ADMETlab 2.0 and toxCSM, as we consider them state of the art given their respective predictive performance for pharmacokinetic and toxicity prediction. The number of data points may vary between the original dataset and the curated version due to the standardization process. In summary, we assessed the Deep-PK pipeline on 53 and 36 endpoints for the ADMETlab 2.0 and toxCSM platforms, respectively.

For the Deep-PK pipeline models, the curated datasets underwent a random split into training, validation and test datasets at a ratio of 8:1:1 after merging across all available databases and standardization. Consequently, Deep-PK encompasses 73 endpoints, involving both 49 classification and 24 regression tasks. All datasets are available for download at https://biosig.lab.uq.edu.au/deeppk/data.

*Analysis of molecular properties*. Using a radar plot, we added an indication of whether users’ query molecules fall within the range of certain FDA-approved drugs’ ADMET range. This range is based on 329 FDA-approved drugs and their predicted ADMET values (see Supplementary Figure S8).

*Prediction confidence*. The reliability of models is pivotal in assessing their credibility, and this is often gauged through prediction probabilities, particularly in the context of classification tasks (38). While this measure is valuable, it may not comprehensively address the coverage of chemical space bridging the training data and query molecules.

In pursuit of a more nuanced understanding of confidence, Deep-PK goes beyond mere prediction probabilities. For both classification and regression tasks, our model assesses whether the query molecule falls within the 95% range of training molecular weight. Additionally, in regression tasks, the model checks whether the predicted values align with the 95% range of training labels. This extended scrutiny offers a more detailed perspective on the model’s confidence, accounting for both the molecular weight and predicted values, thereby providing a richer evaluation of model reliability across various dimensions of chemical space.

### Deep learning

*Graph neural networks*. The core framework of Deep-PK relies on the D-MPNN of Chemprop introduced by Yang *et al.* (39). D-MPNN facilitates information propagation through directed edges, enabling the transmission of crucial information with a specific directionality between nodes. This unique feature empowers D-MPNN to enhance the aggregation of molecular representations by finely controlling the flow of information.

During the training phase, our pipeline employed Matthew’s correlation coefficient (MCC) for classification tasks and *R*^2^ (coefficient of determination) for regression tasks as loss functions. This selection was made to optimize the model’s performance across different tasks. To mitigate batch effects and reduce the risk of obtaining inflated performance values, we implemented an ensemble method, 3-fold cross-validation, which averages and calculates the standard deviation of performance metrics across three models, ensuring a comprehensive and consistent assessment of performance. This approach enhances the robustness of our model evaluations by providing a more reliable measure of predictive accuracy.

*Feature engineering*. In addition to the initial 8 atom (node) features and 4 bond (edge) features adopted from Chemprop, our approach incorporates 216 graph-based signatures (32,40), 37 toxicophore counts (41) and 196 molecular descriptors extracted from RDKit, except 4 descriptors such as ‘MaxAbsPartialCharge’, ‘MaxPartialCharge’, ‘MinAbsPartialCharge’ and ‘MinPartialCharge’, which returned a not-a-number value on several SMILES, as additional molecular (graph) level features. To ensure the efficiency and relevance of the features, we applied a variance threshold of 0.0 to eliminate any non-relevant and non-additive information (42).

*Hyperparameter optimization*. To efficiently search for all 12 individual hyperparameter spaces from the Chemprop pipeline, we employed a Bayesian optimization method with 500 randomly initialized runs and another 500 values for posterior optimizations on the following parameters: the number of message passing iterations (depth), the dropout probability (dropout), activation function, batch size, aggregation, the hidden size of neural network layers, initial learning rate, maximum learning rate, final learning rate, warm-up epochs, the number of feed-forward layers and aggregation normalization. In total, we ran 160 000 optimization runs for 73 Deep-PK, 53 ADMETlab 2.0 and 36 toxCSM endpoints individually.

*GNN interpretability*. The concept of explainability in GNNs has been known to be useful for identifying key parts (subgraphs) of prediction. We use this substructure highlighting of Chemprop to provide ideas like what sites should be kept, changed or removed from queries in terms of each endpoint and across those that shared the same explainability (Supplementary Figures S9 and S10).

*Optimization*. The optimization of Deep-PK generates up to 100 samples from a query molecule by adding, replacing or removing substructures using the MultI-constraint MOlecule SAmpling (MIMOSA) method (43). We implemented a pre-trained model for optimizing the quantitative estimate of drug-likeness (QED) (44), which is one of the key features of drug discovery.

## Web server

Deep-PK was developed using Bootstrap 3.3.7 and Flask 1.0.2 and hosted on an Apache 2 Ubuntu server. This web server is freely available at https://biosig.lab.uq.edu.au/deeppk.

### Input

Deep-PK can be used to assess the pharmacokinetic and toxicity properties of small molecules based on ADMET and, also, general properties. As shown in Supplementary Figure S1, Deep-PK accepts four types of inputs: (i) a single SMILES string; (ii) a SMILES file containing a set of small molecular compounds (up to 2000 molecules); (iii) a structural data file (SDF) covering a list of molecular structures (up to 2000 molecules); and (iv) a single molecular drawing, where its respective SMILES is retrieved from the drawn molecule structure. These are the most standard formats in small molecule research, commonly used in their respective web servers. Example files and a help page to guide users are provided at https://biosig.lab.uq.edu.au/deeppk/prediction and https://biosig.lab.uq.edu.au/deeppk/help, respectively. Users can choose which ADMET prediction mode they want to make their compound analysis on, including running everything all at once.

### Output

The web server encompassing Deep-PK stresses its four main prediction outputs: (i) the prediction results page, where the pharmacokinetic, toxicity and general small molecule properties are displayed after all selected models have their outcomes completed (Supplementary Figure S2); (ii) the focus page, where the predictions of each molecule are better detailed and explained with confidence values and interpretations based on the literature (Supplementary Figure S3); (iii) the analysis page, where each molecule that had their ADMET properties predicted can be further analysed in terms of general properties, drug-likeness and predictive substructure importance (Supplementary Figures S4–S10); and (iv) the optimization page, where the given query molecule is optimized, aiming to improve the pharmacokinetic and toxicity properties, which are of interest to the target user or researcher (Supplementary Figure S11). Whereas the results for the prediction and optimization pages can be downloaded in a CSV (comma-separated value) file, the results of the analysis page are retrieved in a ZIP file.

### Application programming interface

The Deep-PK integrates an application programming interface (API) to facilitate the development, validation and testing of cheminformatics and drug discovery analytical pipeline. Each submission in Deep-PK’s API has a unique string identifier, which can be used to retrieve the predictive results after the web server processing. It is worth noting that these results are also available through the web server prediction results page. Deep-PK’s API allows the use of SDFs, SMILES files and SMILES strings. When the predictions are completed, Deep-PK outputs only standard tabular results in a JavaScript object notation format. The documentation (in both Python and Curl languages) and examples of how to use Deep-PK’s API are available at https://biosig.lab.uq.edu.au/deeppk/api_docs.

### Processing times

Deep-PK has been proposed to serve as a scalable platform for predicting a diverse range of pharmacokinetic and toxicity properties of small molecules. While assessing Deep-PK’s capabilities, we estimated that it takes up to 15 min to predict all 73 ADMET properties for a single molecule after the prediction processing work starts—i.e. after the respective web server job is released from the queue to be processed. Furthermore, the processing time for optimizing a single molecule and providing the predictions for the resultant 100 optimized molecules is estimated to be 20 min after being released from the optimization queue. Finally, Deep-PK takes up to 55 min to analyse the characteristics of each molecule, considering this is the first job to be processed in the analysis queue. The small molecule analysis includes the substructural explanations per endpoint, which we consider as the current processing bottleneck for Deep-PK. Future work involves improving the efficiency of the molecule’s explanation component.

## Validation

The performance of Deep-PK models/pipelines was thoroughly compared with available tools. Since those tools were trained and tested on different datasets that are difficult to obtain, the metrics, MCC and *R*^2^ for classification and regression tasks, for each approach were collected from their original papers. To simplify and enhance visualization, the 73 results were categorized into six types of target predictions: absorption, distribution, metabolism, excretion, toxicity and property. These predictions were further divided into two task types: classification and regression.

### Benchmarking across available tools

*ADMETlab 2.0*. The comprehensive evaluation of the Deep-PK pipeline (Figure 1), encompassing feature extraction to hyperparameter optimization, was compared with ADMETlab 2.0, a cutting-edge pharmacokinetic and toxicity prediction tool. The assessment covered 53 endpoints, comprising 40 classification and 13 regression tasks, providing a thorough examination of the model’s performance across diverse targets.

To simplify the presentation of results, both MCC and *R*^2^ metrics, ranging from 0 to 1 for classification and regression tasks, were merged in Supplementary Figure S12. Notably, Deep-PK exhibited enhanced performance (the performance increase is >0) across 46 endpoints, showcasing its highest performance gap of 0.43 on ‘NR-A’, while demonstrating comparatively lower performance on 5 endpoints with the lowest performance gap of −0.082 (MCC) from ‘oral bioavailability 30%’ (see Supplementary Figure S12).

Analysing the average performance across each category, Deep-PK showed the most notable improvement in metabolism achieving an average performance increase of +0.105 across 10 metabolism endpoints (see Supplementary Table S2). Adopting a categorization strategy where performance changes within ±0.1 are considered no improvement, those exceeding 0.1 are deemed reasonable improvement and surpassing 0.2 are considered significant improvement, Deep-PK achieved significant improvement in 4 models, reasonable improvement in 17 models and maintained stability in 32 models with Δ ±0.1. This nuanced breakdown provides a nuanced understanding of the Deep-PK model’s performance across various pharmacokinetic endpoints.

*toxCSM*. In the assessment of 36 toxCSM toxicity profiles (Supplementary Figure S13), Deep-PK demonstrated enhanced performance across 30 endpoints, notably exhibiting a substantial improvement in MCC (0.39) for ‘NR-ER’—a reasonable enhancement comparable to the earlier ADMETlab 2.0 benchmark (MCC improvement of 0.13). The overall performance averages, encompassing MCC and *R*^2^, were 0.58 for toxCSM and 0.71 for Deep-PK.

By employing a performance delta (Δ) classification, where ±0.1 signifies no/weak improvement, >0.1 and <0.2 indicates reasonable improvement and ≥0.2 denotes significant improvement, Deep-PK achieved 7 significant improvements, 10 reasonable improvements and 19 instances where no/weak improvement was observed (see Supplementary Table S3).

### Performance on Deep-PK datasets

As we confirmed that the Deep-PK pipeline is powerful enough to improve the performance on most pharmacokinetic, toxicity and general property endpoints, we applied the same workflow on the Deep-PK datasets, which were merged and curated across four databases.

The performance of Deep-PK on this curated dataset was subsequently compared with other available tools (see Supplementary Table S4 and Supplementary Figures S14 and S15), albeit without employing identical training and test datasets. It is important to note that such comparisons may not be directly equitable. Nevertheless, they provide valuable insights into the performance enhancements facilitated by our novel approach and the integration of new datasets.

By comparing Deep-PK with other available methods (maximum available methods versus Deep-PK), we observed significant improvements in 6 instances (up to an MCC increase of +0.43) on ‘renal excretion (OCT2)’, alongside reasonable enhancements in 15 cases (MCC increase of +0.1 or greater) and weaker improvements across 38 endpoints. Consistent with previous benchmark tests, the Deep-PK pipeline demonstrated a significant performance boost in the excretion and metabolism category, achieving an average MCC increase of +0.10 and +0.07 across 13 metabolism and 3 excretion endpoints, respectively.

## Discussion

The prediction of pharmacokinetic and toxicity properties is a key step during the drug screening and lead optimization processes. As more data and machine learning algorithms are available, more accurate predictions have been available (25,32–35). Due to the nature of the difficulty of collecting data, many tools have not been thoroughly benchmarked or did not include their predictors in a single platform for better usability. Although GNN has empowered the field of pharmacokinetic and toxicity predictions with its benefit of featurizing from the molecule itself giving the state-of-the-art performance (25,35), the lack of performance comparison across different approaches has been a limit for using or comparing each other.

We anticipate that Deep-PK’s predictive models, which are anchored in the chemical landscape illustrated in Supplementary Figure S16, possess a degree of generalizability that should translate into robust performance when applied to novel molecules. Our analysis through principal component analysis, utilizing both MACCS keys (Supplementary Figure S16A) and Morgan fingerprints (Supplementary Figure S16B), demonstrates that the structural diversity of 2.3 million compounds from ChEMBL (ChEMBLdb 33) is well represented, even with a dataset size 20 times smaller than Deep-PK. Remarkably, the chemical spaces of FDA-approved drugs (DrugBank) fall within the coverage of Deep-PK, underscoring the potential utility of our models for targeting FDA approval pathways.

However, it is crucial to acknowledge that the accuracy of predictions may be constrained by the inherent limitations of available assay data, particularly when users submit compounds that are markedly different from those in the training dataset. While our focus does not extend to ‘zero-shot learning’, we expect a certain level of similarity between user queries and the datasets used to train, validate and test Deep-PK models. Moreover, to bolster confidence in our predictions, Deep-PK furnishes additional confidence values for each outcome (Supplementary Figure S3).

By integrating molecular properties as graph-level features, Deep-PK significantly enhanced prediction performance across two benchmark datasets (i.e. ADMETlab 2.0 and toxCSM), notably achieving improvements of >0.43 and 0.39 in MCC values for NR-AR and NR-ER, respectively. These advancements underscore Deep-PK’s potential as a robust screening platform for nuclear hormone receptors, signifying its potential utility in identifying endocrine-disrupting chemicals, which are pivotal in various fields and types of drugs.

Additionally, it is noteworthy that assessing the variation of model performance across 3-fold cross-validations, which maintain identical hyperparameter configurations but undergo dataset shuffling with different random seeds, can be crucial in gauging the reliability of deep learning models. For example, Supplementary Figure S17 illustrates significant performance fluctuations observed during 3-fold cross-validations of the Cyp1a2 substrate, particularly when models exhibit lower performance. This statistical insight can instill greater confidence in the models, especially when their performance is naturally constrained from achieving higher levels of accuracy. We have confirmed that Deep-PK models demonstrate consistent performance across all 73 endpoints, further enhancing confidence in their reliability (Supplementary Table S4).

However, we observed some performance degradation using Deep-PK datasets, particularly on four endpoints (Supplementary Table S4), where the performance change (Δ) ranged from −0.15 to −0.58. The possible main factor behind this performance drop is data corruption, as evidenced by the contrast in performance. For example, while Deep-PK achieved improved performance on Marine Tox (fathead minnow) with MCC values of 0.767 and 0.601 on ADMETlab 2.0 and toxCSM datasets, respectively, it experienced a significant drop to an MCC of 0.16. This suggests a possible data corruption issue in the process of merging the two datasets. Consequently, we opted to select the best-performing model across three versions of our datasets.

It is crucial to distinguish between model credibility and performance, as they represent distinct facets. While performance confidence often focuses on metrics like classification probability, this approach has inherent limitations, particularly regarding its applicability to classification tasks exclusively. However, for reliable predictions, it is imperative that query molecules inhabit similar chemical spaces and label ranges to the training data. To address this, Deep-PK effectively incorporates additional confidence tags, such as ranges of molecular weights and labels (Supplementary Figure S3), empowering users to interpret prediction results with greater certainty.

Furthermore, GNNs offer a unique advantage by encapsulating molecular features at node and edge levels, facilitating information propagation across different layers. Leveraging graph-level features enables Deep-PK to identify key molecular regions influencing predictions, shedding light on critical insights. Notably, Deep-PK not only highlights the most influential subgraphs within a query molecule (Supplementary Figure S9) but also identifies common subgraphs among categories (Supplementary Figure S10). This capability proves invaluable, particularly in multifaceted categories like metabolism and toxicity, guiding targeted lead optimization efforts. Additionally, Deep-PK provides an indication of whether users’ query molecules fall within the range of certain properties, particularly regression properties, such as FDA-approved drugs’ ADMET range. This range is based on 329 FDA-approved drugs and their predicted ADMET values (see Supplementary Figure S8).

The goal of drug discovery optimization is to enhance molecules based on ADMET endpoints. To further enhance molecules, Deep-PK introduces an optimization protocol that modifies substructures, bridging a gap in pharmacokinetic prediction platforms that typically focus solely on value prediction. Using the MIMOSA library, Deep-PK generates 100 new molecules from a query molecule with an improved QED. These molecules are then evaluated for pharmacokinetic and toxicity profiles, highlighting any changes in endpoints resulting from the optimization process. While Deep-PK’s optimization function generates novel molecules with the potential for improved ADMET properties, it is crucial to acknowledge that molecule generation primarily relies on log *P* and QED. Consequently, the outcomes of optimization may not consistently reflect enhanced ADMET characteristics. Nevertheless, the optimized molecules typically show heightened QED values and maintain compatibility across various chemical spaces, attributable to the comprehensive dataset utilized in their training.

In summary, Deep-PK emerges as an invaluable screening and optimization platform for the drug discovery process, offering deep analysis based on state-of-the-art performing GNN models and rapidly optimizing new molecules tailored for drug candidates. By doing so, it significantly reduces costs and time associated with unnecessary *in vitro* or *in vivo* pre-clinical tests, while also mitigating potential risks inherent in these tests.

## Supplementary Material

gkae254_Supplemental_File

## Data Availability

The curated ADMET training, validation and test datasets representing the pharmacokinetic and toxicity assays are available at https://biosig.lab.uq.edu.au/deeppk/data. The dataset characteristics are properly explained at https://biosig.lab.uq.edu.au/deeppk/theory.
