# Supplementary material for: Deep-PK: deep learning for small molecule pharmacokinetic and toxicity prediction
Source: Nucleic Acids Res. 2024 Apr 18;52(W1):W469–75. doi: 10.1093/nar/gkae254 (PMC11223837; doi:10.1093/nar/gkae254)
Supplement: gkae254_Supplemental_File [file gkae254_supplemental_file.docx]

**SUPPLEMENTARY DATA**

**Deep-PK: deep learning for small molecule pharmacokinetic and toxicity prediction**

Yoochan Myung^1,2^, Alex G.C de Sá^1,2,3,#^, David B. Ascher^1,2,3,#^

^1^ School of Chemistry and Molecular Biosciences, The Australian Centre for Ecogenomics, The University of Queensland, Brisbane, Queensland, 4072, Australia

^2^ Computational Biology and Clinical Informatics, Baker Heart and Diabetes Institute, Melbourne, Victoria, 3004, Australia

^3^ Baker Department of Cardiometabolic Health, The University of Melbourne, Parkville, Victoria, 3010, Australia

#To whom correspondence should be addressed to D.B.A. Tel: +61 7 336 53891; Email: [d.ascher@uq.edu.au](mailto:d.ascher@uq.edu.au).. Correspondence may also be addressed to A.G.C.S. at [Alex.deSa@baker.edu.au.](mailto:Alex.deSa@baker.edu.au)

**Figures**


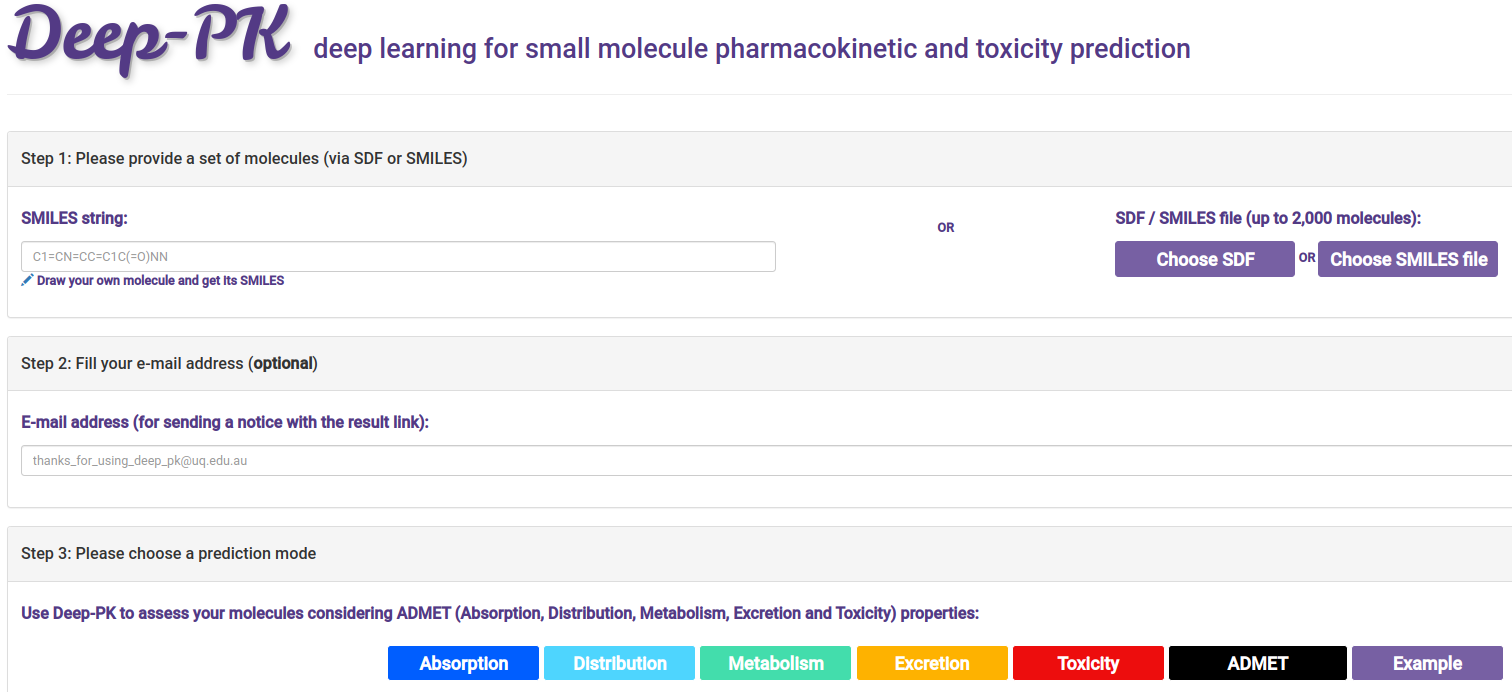


**Figure S1. The submission page for Deep-PK web server.** The submission page offers four types of inputs: SMILES string, SMILES file, SDF file, and molecular drawing. Users have the option to provide their email address to receive a link with the results after the processing of the molecule(s). Deep-PK offers prediction modes for all ADMET categories separately as well as conjunctively.


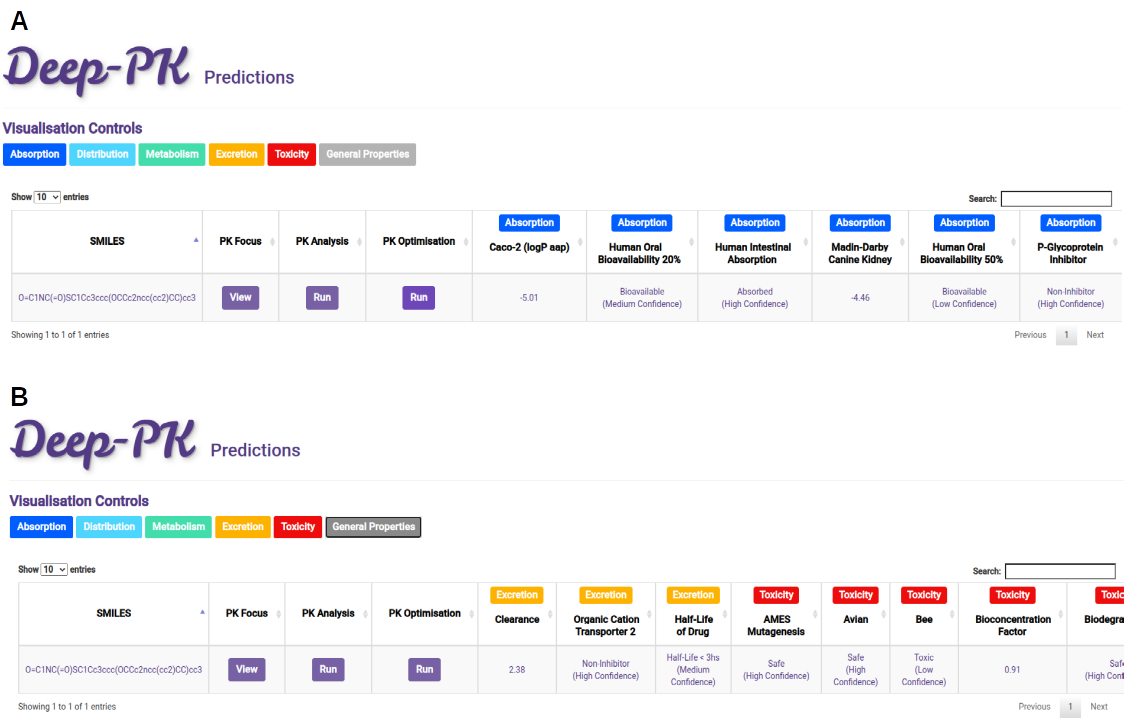


**Figure S2. The result page for Pioglitazone.** The results for the anti-diabetic medication Pioglitazone, highlighting predicted (A) Absorption and (B) Excretion and Toxicity properties.


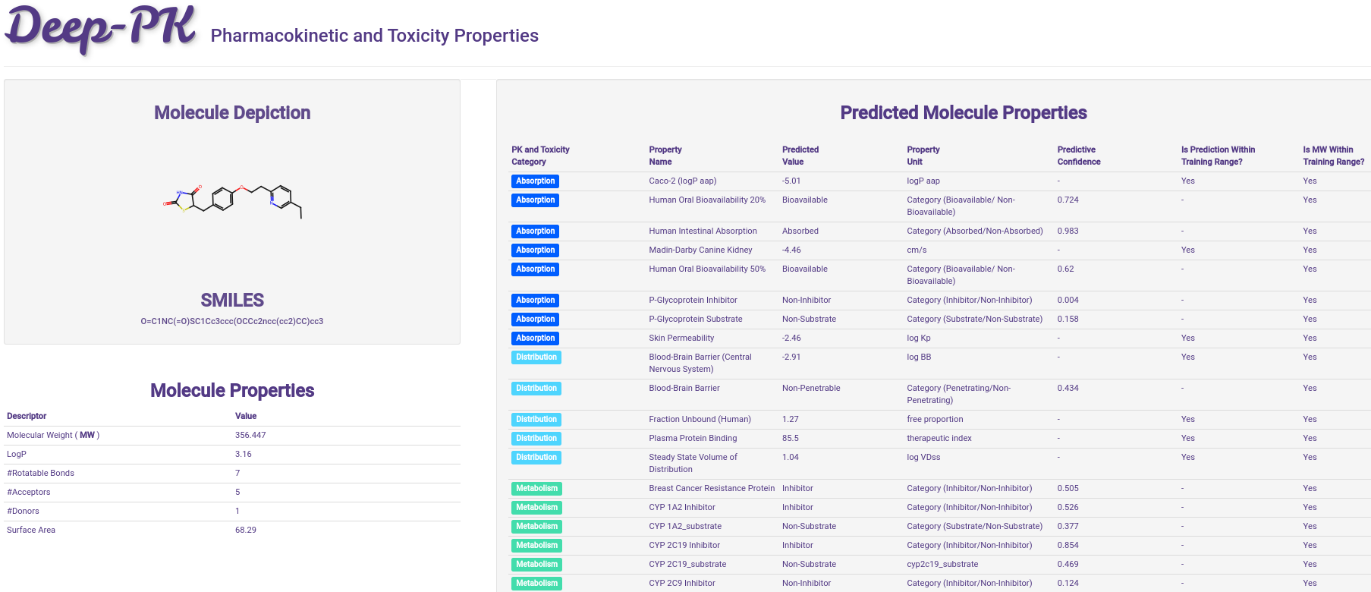


**Figure S3. The PK Focus page for Pioglitazone**. The Focus page highlights the molecule depiction, SMILES, and properties. This page also displays the predicted properties alongside their confidence levels. Additionally, it includes binary outputs for regression tasks, indicating whether the molecule's weight and predictions fall within the training sets of the respective models.


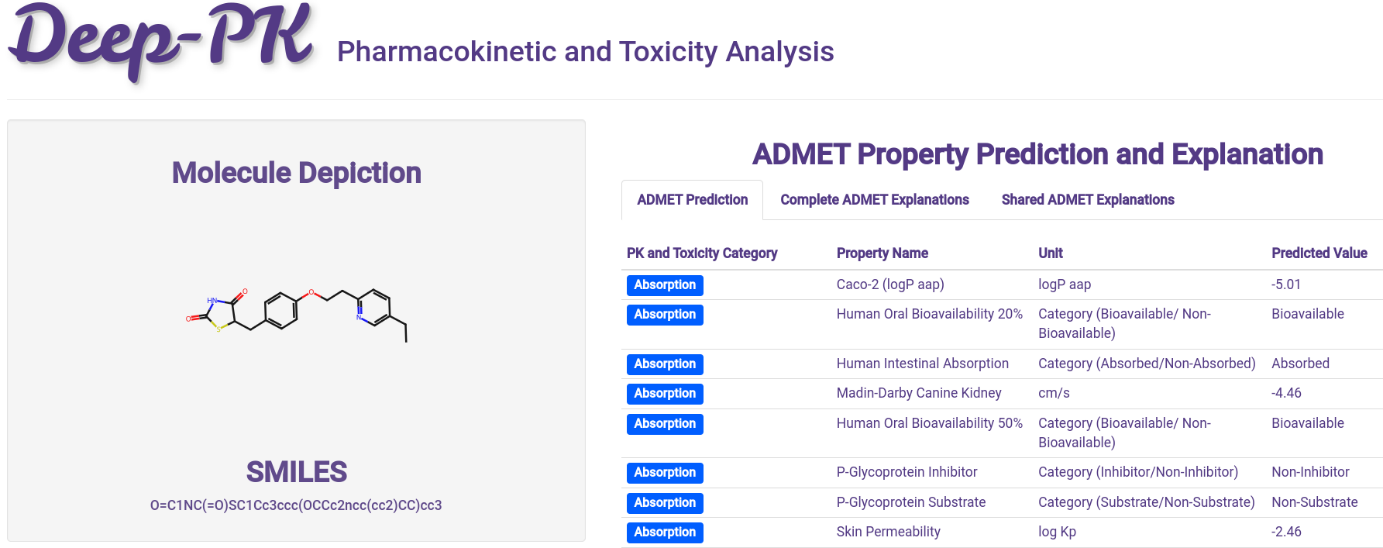


**Figure S4. The PK Analysis page for Pioglitazone (Part 1).** The Analysis page presents a molecule depiction, and ADMET predictions of a query molecule are displayed at the top of the analysis page.


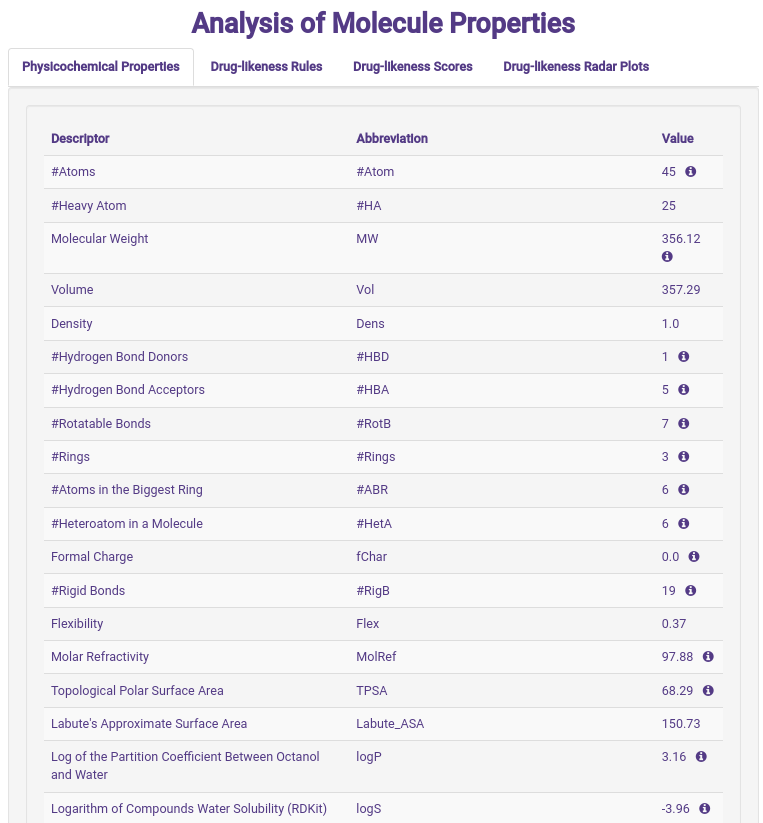


**Figure S5. The PK Analysis page for Pioglitazone (Part 2).** The Analysis of Molecular Properties provides a range of molecular properties, bringing information about the acceptability of these properties for drug-likeness rules (indicated with the tooltip).


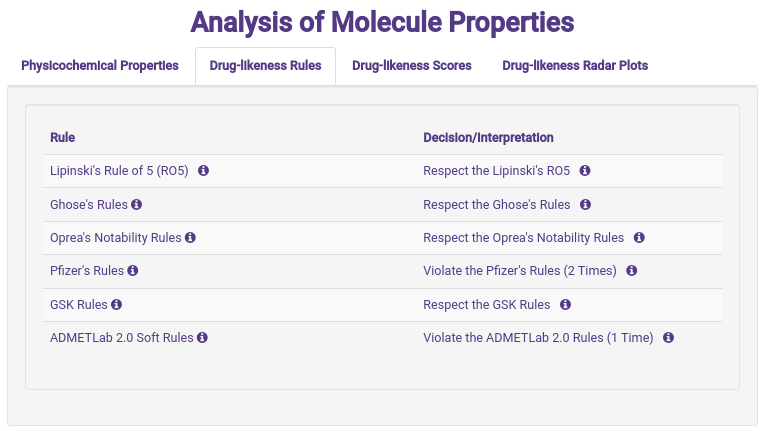


**Figure S6. The PK Analysis page for Pioglitazone (Part 3).** The Analysis page indicates if the molecule complies drug-likeness rules specifically.


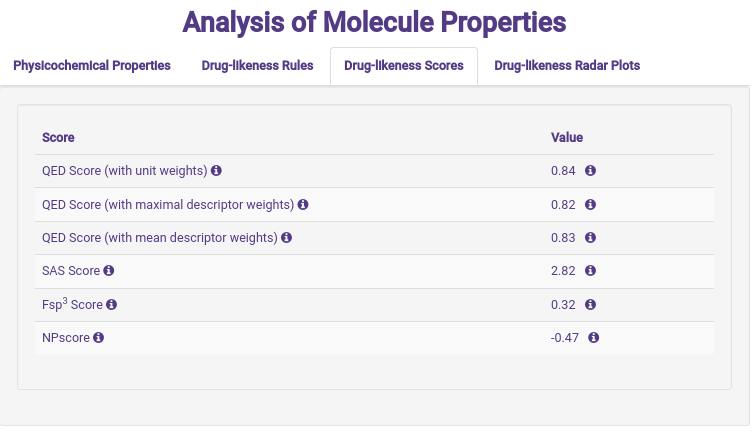


**Figure S7. The PK Analysis page for Pioglitazone (Part 4).** The Analysis page presents drug-likeness scores, mapping with the information tooltip if the reached values are appropriate or not.


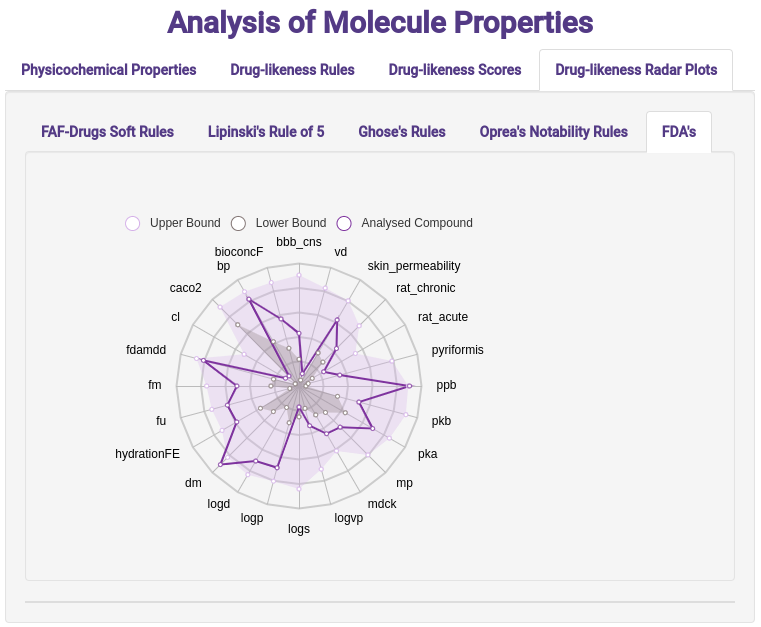


**Figure S8. The PK Analysis page for Pioglitazone (Part 5).** Different drug-likeness radar plots are also shown on the Analysis page, including FAF-Drugs Soft Rules, Lipinski's Rule of 5, Ghose’s Rules, Oprea’s Notability Rules and FDA’s Approved Drugs.


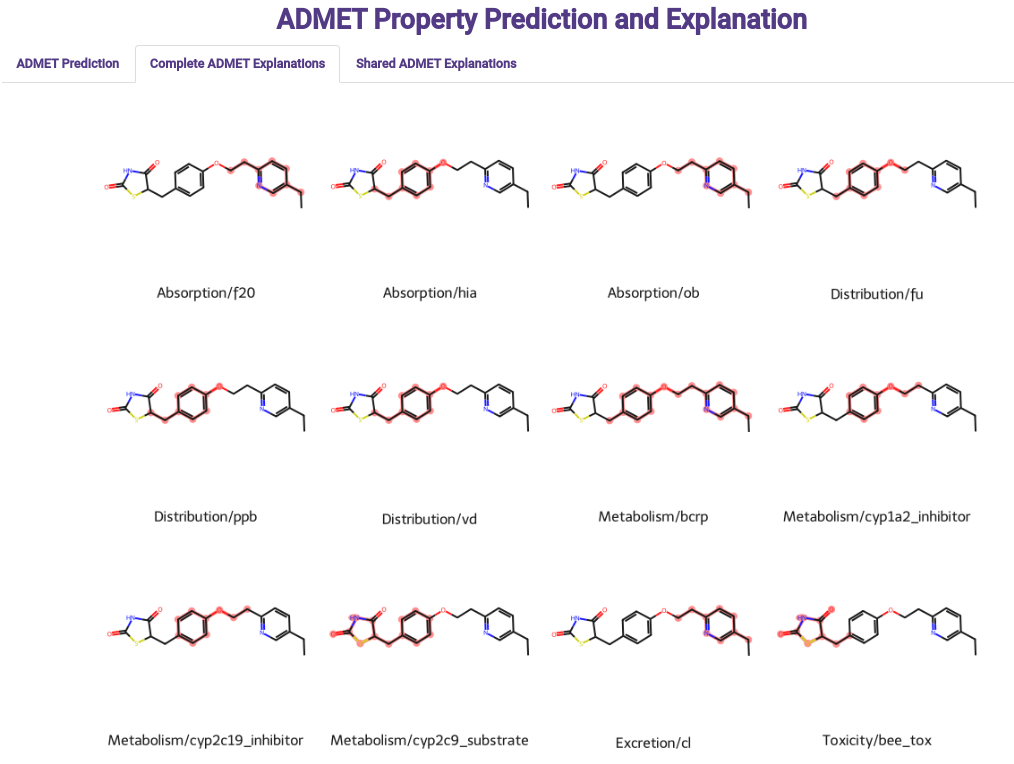


**Figure S9. The PK Analysis page for Pioglitazone (Part 6).** Substructure importances, elucidating the most relevant parts for the predictions regarding the endpoints, are highlighted within the molecule's structure on the Analysis page.


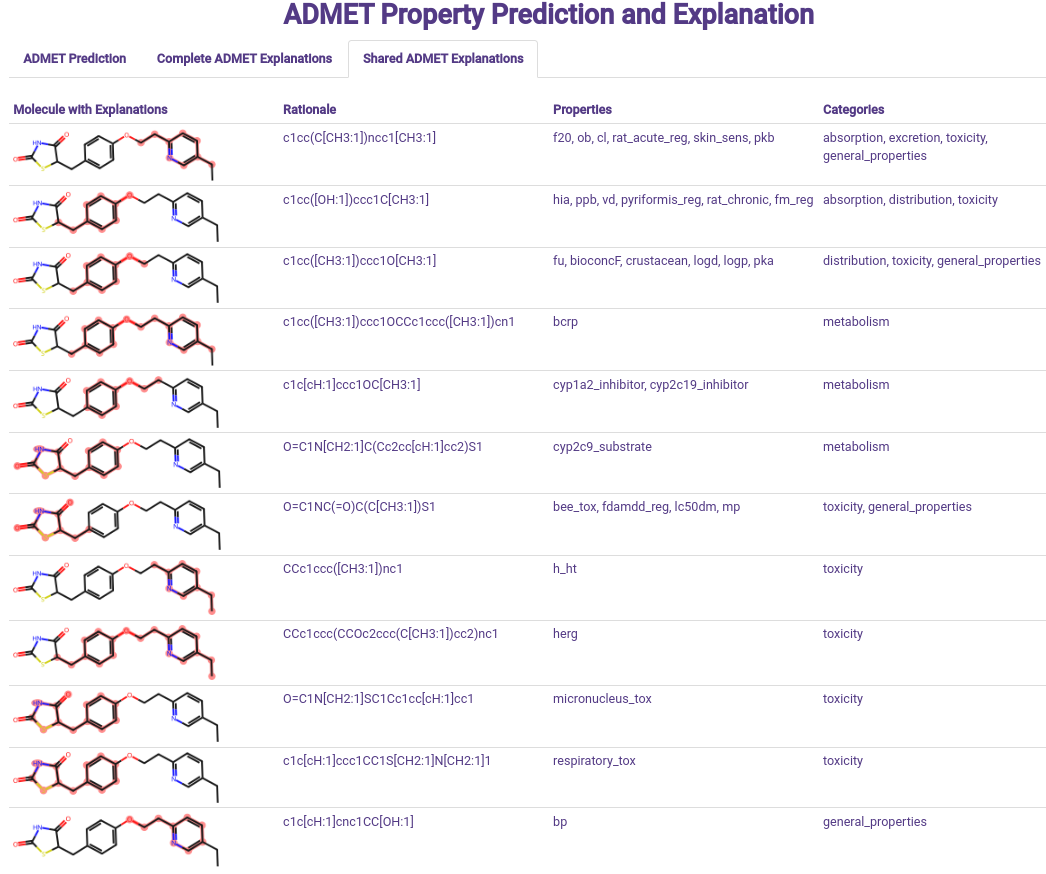


**Figure S10. The PK Analysis page for Pioglitazone (Part 7).** Shared substructure importances across the endpoints are also mapped and defined in a table, facilitating comparisons of the degree of similarity of the predictions across the different ADMET endpoints and categories.


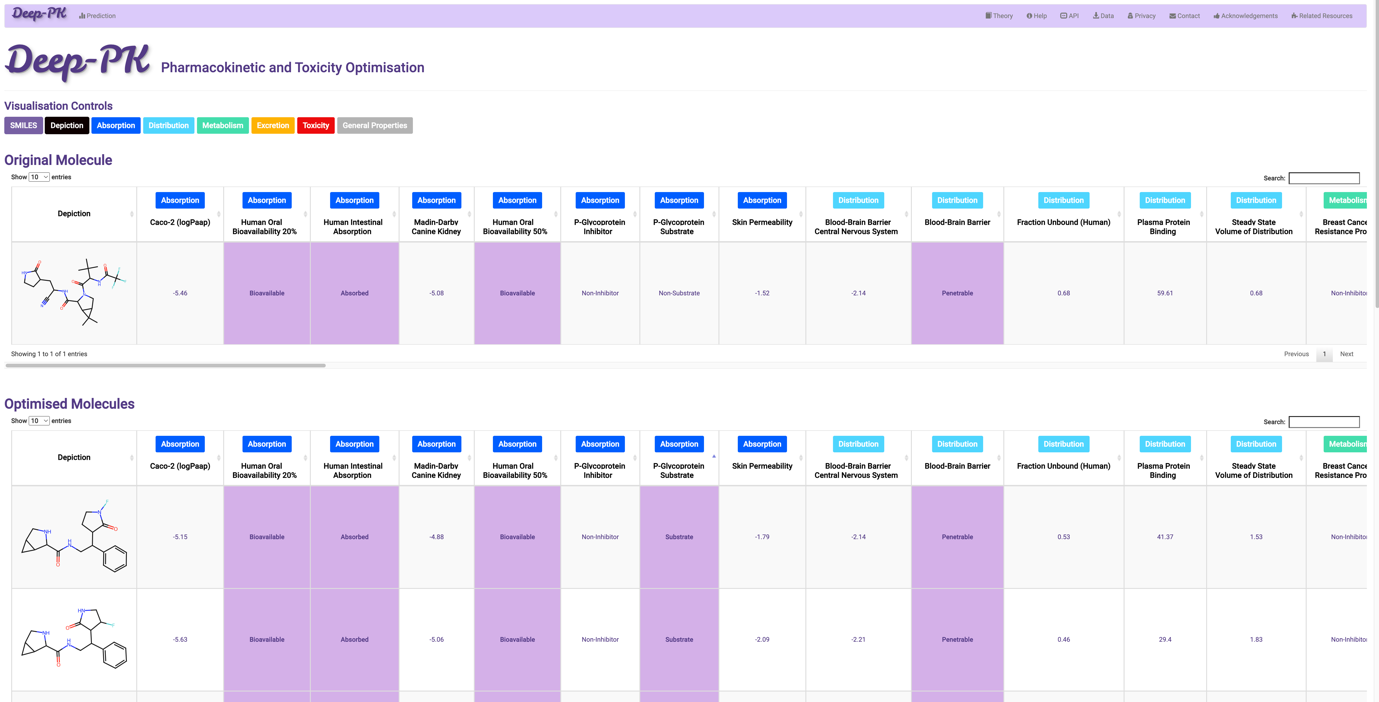


**Figure S11. The Optimisation page for Paxlovid.** The Optimisation page demonstrates Deep-PK’s capabilities to create molecules with different properties, and to compare the results one by one across the different endpoints and predictions.


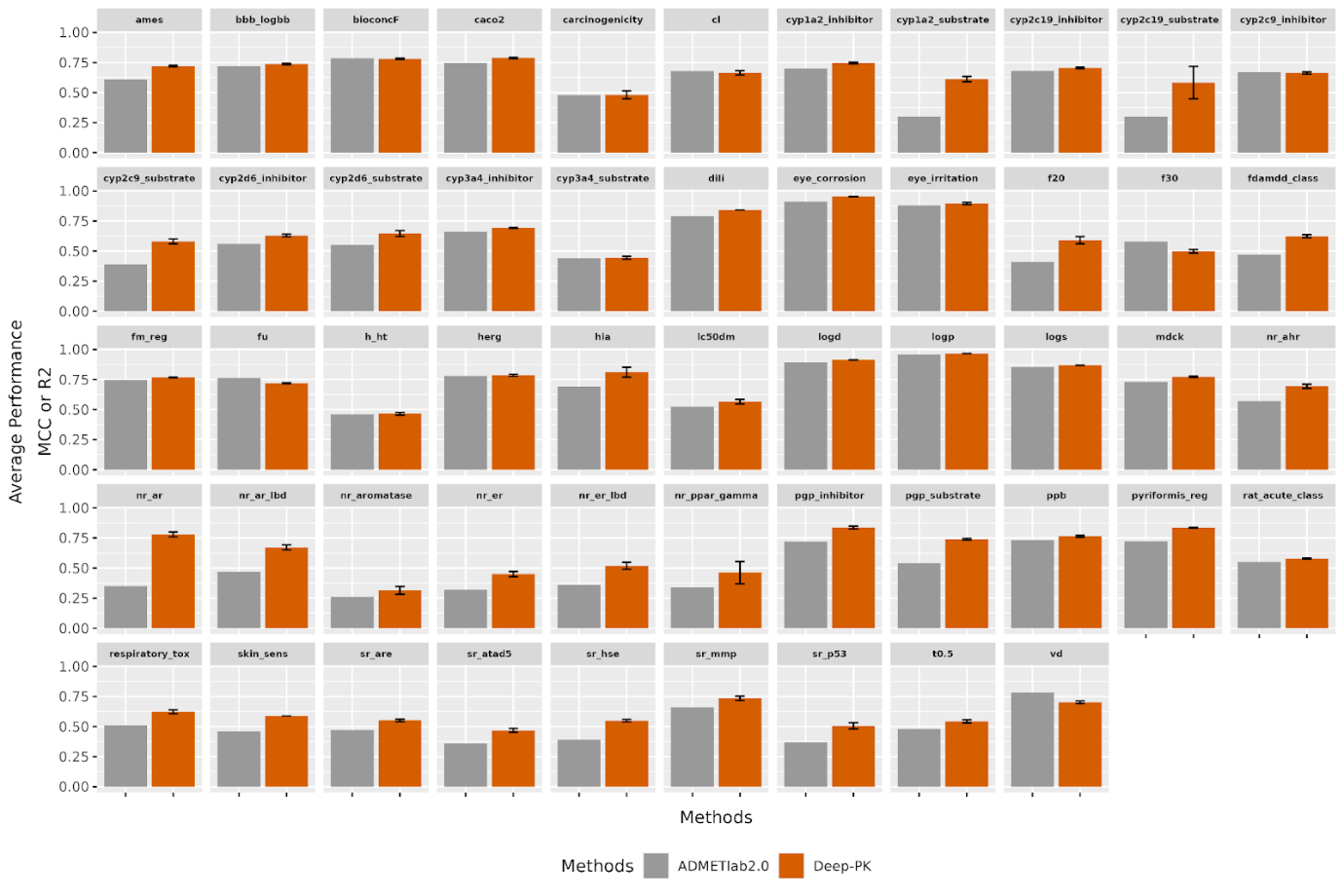


**Figure S12. Deep-PK benchmark on ADMETlab 2.0.**  The metrics (MCC and R^2^) on 53 endpoints in ADMETlab 2.0 were collected from the publication(1). Deep-PK's performance was computed through averaging across a 3-fold cross-validation. The error bar, indicative of the consistency of model performance, displays the standard deviation across three models.


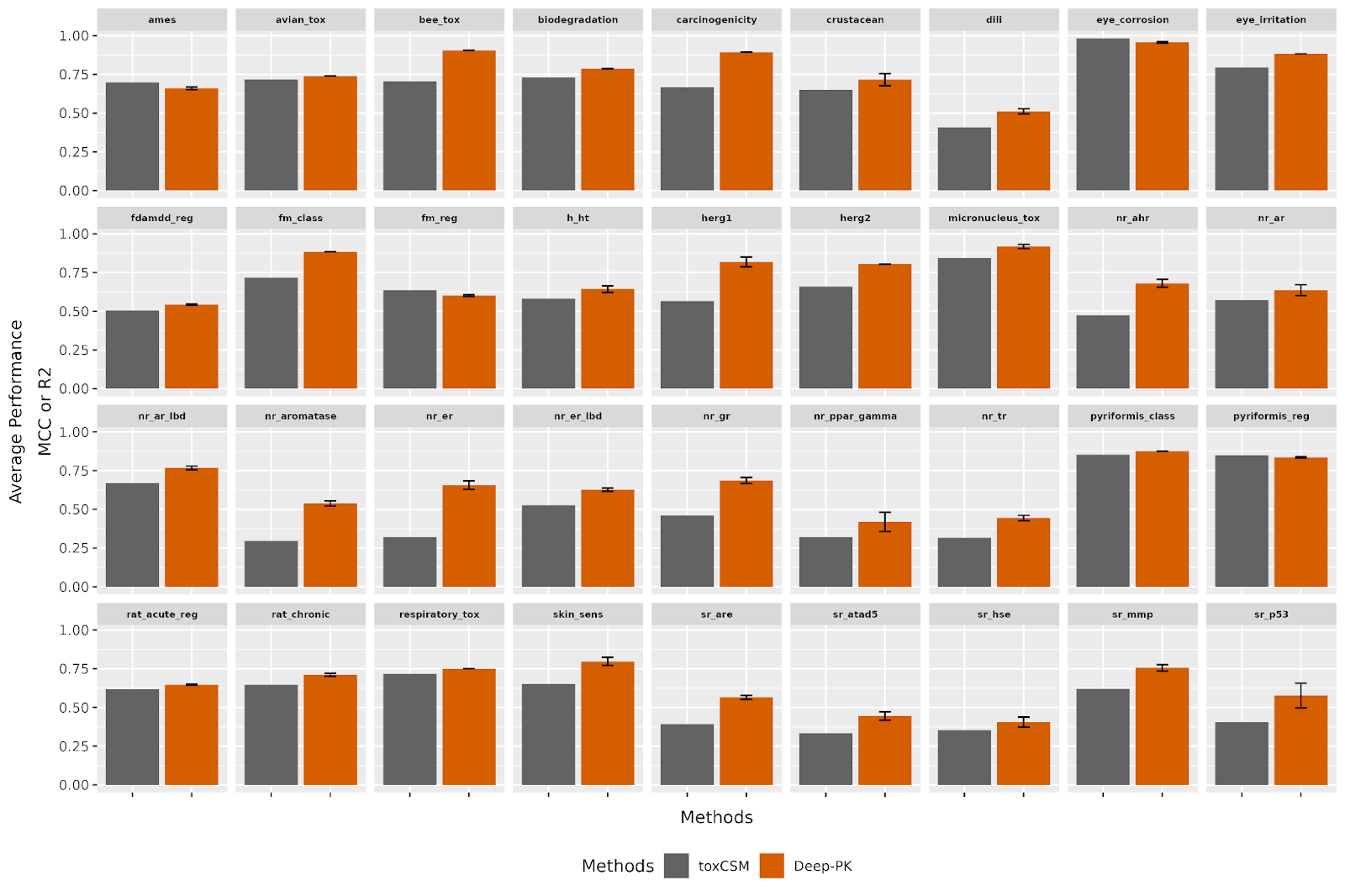


**Figure S13. Deep-PK benchmark on toxCSM.**  The metrics (MCC and R^2^) on 36 endpoints in toxCSM were collected from the publication(2). Deep-PK's performance was computed through averaging across a 3-fold cross-validation. The error bar, indicative of the consistency of model performance, displays the standard deviation across three models.


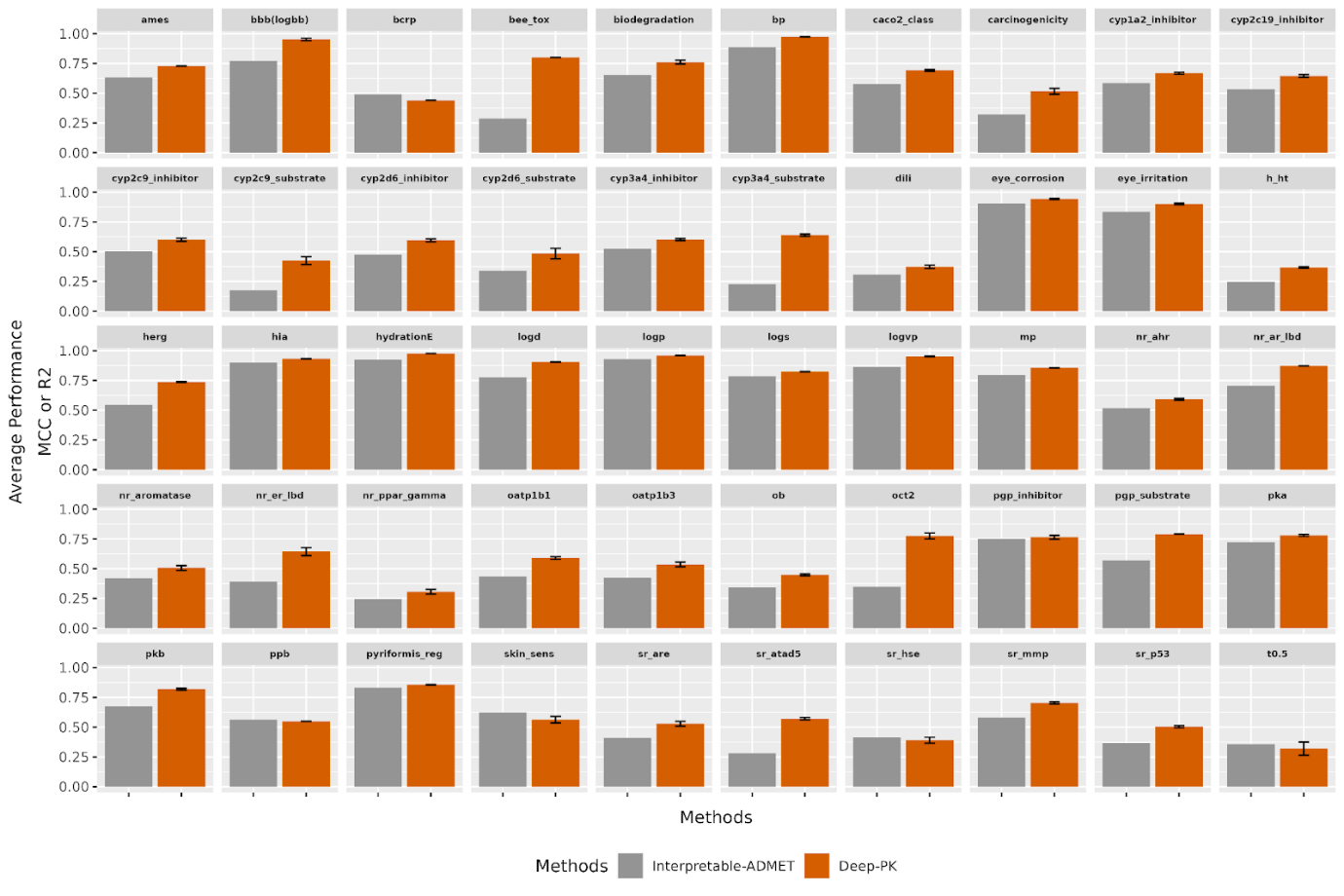


**Figure S14. Performance comparison between Deep-PK and Interpretable-ADMET.**  The best test metrics (MCC and R^2^) for 50 endpoints in Interpretable-ADMET were collected from the publication(3). Deep-PK's performance was evaluated using a 3-fold cross-validation and the results were averaged. The error bars represent the standard deviation across three models, reflecting the consistency of performance. Notably, Deep-PK and Interpretable-ADMET were trained and tested on distinct datasets.


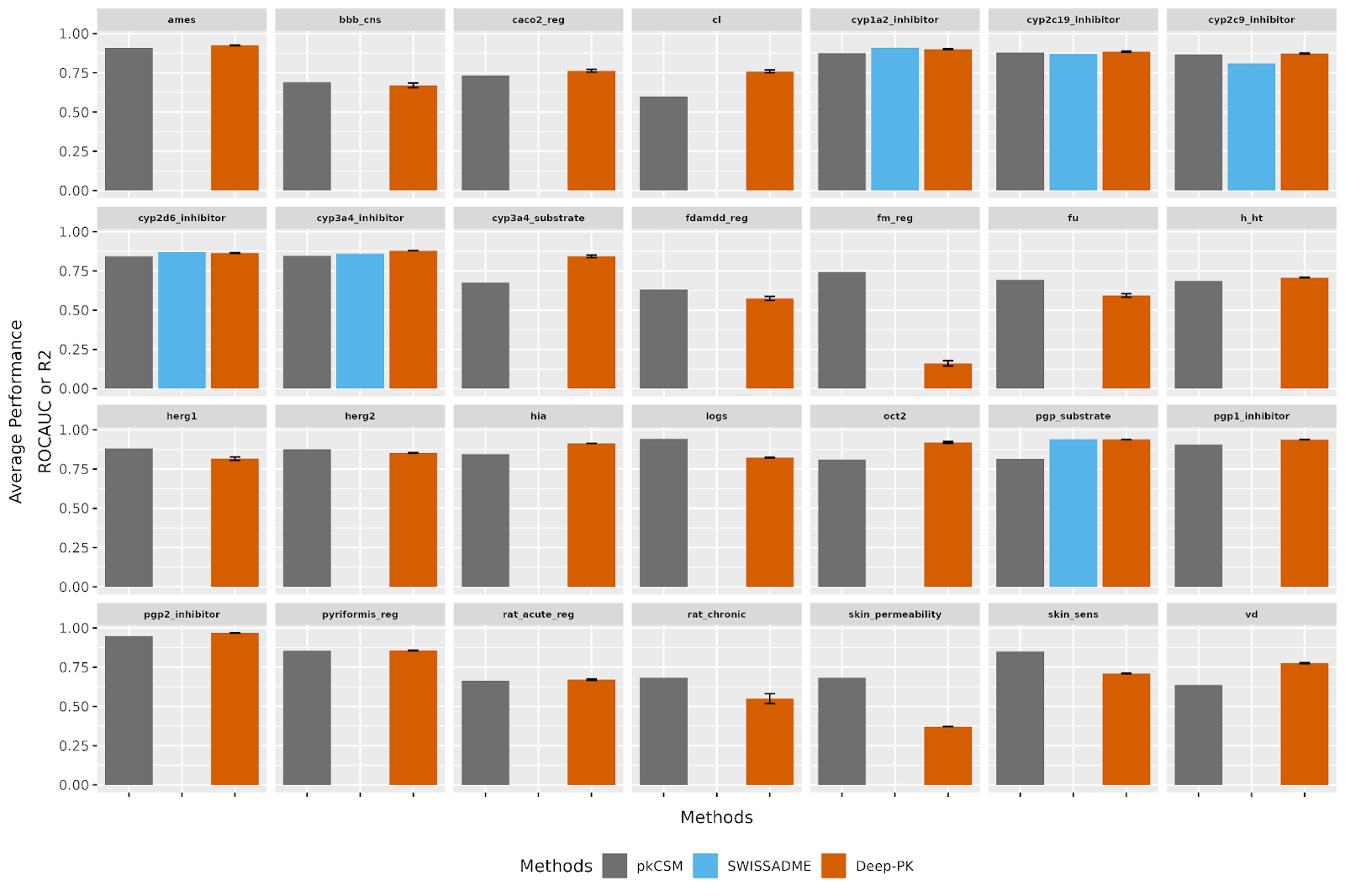


**Figure S15. Performance comparison among pkCSM, SwissADME, and Deep-PK.** Test metrics (ROCAUC and R^2^) for pkCSM(4)and SwissADME(5) endpoints were sourced from their respective publications. Deep-PK's performance was assessed using a 3-fold cross-validation, with results averaged across folds. The error bars denote the standard deviation across three models, indicating the consistency of performance. It's important to note that all tools were trained and tested on separate datasets.


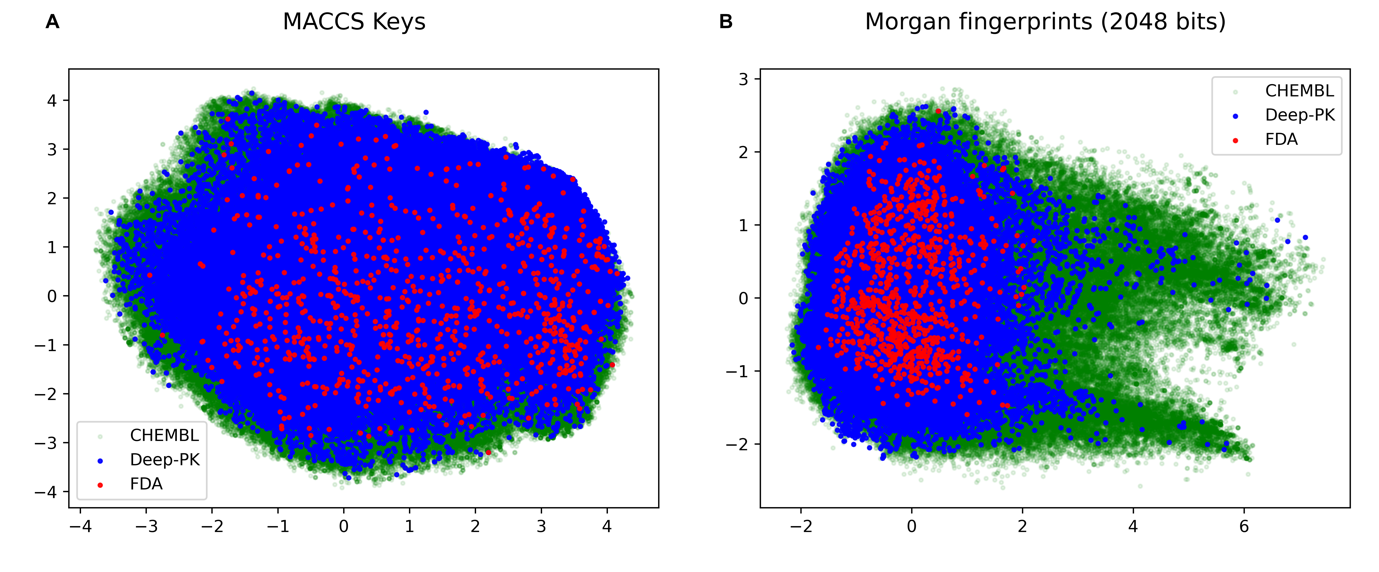


**Figure S16. Comparison of chemical space across three datasets**. The structural diversity of 2,372,673 ChEMBL (ChEMBLdb 33), 116,470 Deep-PK and 868 FDA-approved molecules (DrugBank) was assessed with PCA analysis using (A) MACCS Keys and (B) Morgan fingerprints. MACCS Keys and Morgan fingerprints were generated using the MACCSkeys and GetMorganFingerprintAsBitVect (nBits=2045, radius=2) functions, respectively, from Rdkit-v2022.03.5. The numbers of molecules across three databases were reduced after the standarisation process implemented for the Deep-PK platform.


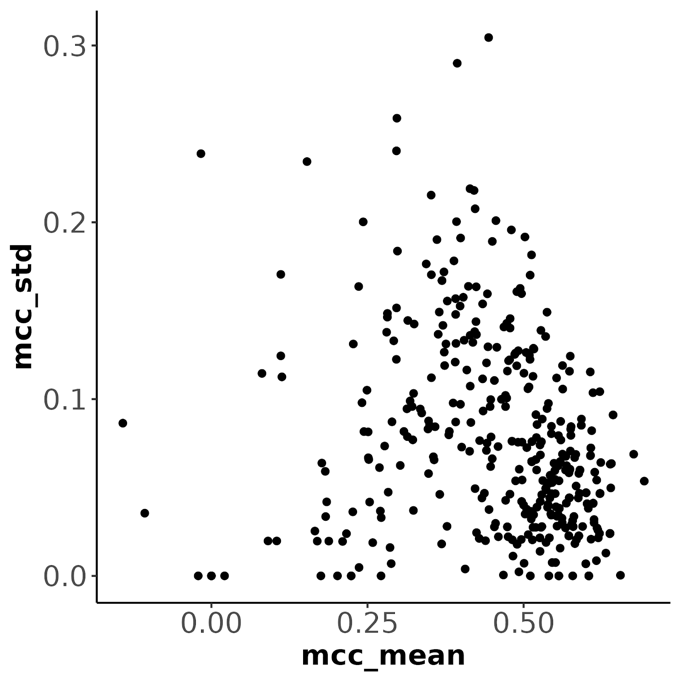


**Figure S17. Scatter plot of hyperparameter optimisation results for Cyp1a2_substrate.** The plot illustrates the average performance of the Deep-PK model on 3-fold cross-validation and its corresponding standard deviation. As depicted, there are fewer variations in cross-validation performance as the model's performance improves.

**Tables**

**Table S1. Details of Deep-PK Dataset.** This table provides an overview of the Deep-PK dataset, including the units and interpretations of each endpoint in the respective columns. Additionally, it outlines the minimum and maximum ranges of molecular weight and label for the corresponding training dataset. The term 'N/A' indicates that the dataset type is categorical.

| **Category** | **Name** | **Type of Task** | **Min_MW** | **Max_MW** | **Min_Label** | **Max_Label** |
| --- | --- | --- | --- | --- | --- | --- |
| Absorption | Intestinal permeability (Caco2) logP | Regression | 40.031 | 1084.473 | -7.72 | -3.75 |
| Absorption | Oral bioavailability 20% | Classification | 89.048 | 499.265 | N/A | N/A |
| Absorption | Intestinal permeability (MDCK) | Regression | 89.048 | 499.265 | -7.12 | -2.45 |
| Absorption | Oral bioavailability 50% | Classification | 89.048 | 499.265 | N/A | N/A |
| Absorption | Drug efflux pump inhibitor (pgp_inhibitor) | Classification | 40.031 | 1084.473 | N/A | N/A |
| Absorption | Drug efflux pump substrate (pgp_substrate) | Classification | 73.089 | 707.530 | N/A | N/A |
| Absorption | Intestinal absorption | Classification | 61.992 | 1084.473 | N/A | N/A |
| Absorption | Skin permeability | Regression | 89.048 | 497.204 | -5.52 | -0.69 |
| Distribution | BBB (CNS) | Regression | 89.048 | 497.204 | -5.70 | -0.70 |
| Distribution | BBB (logBB) | Classification | 32.026 | 1084.473 | N/A | N/A |
| Distribution | Fraction unbound in human plasma | Regression | 40.031 | 1084.473 | 0.00 | 3.00 |
| Distribution | Plasma protein binding | Regression | 32.026 | 1084.473 | 0.00 | 100.00 |
| Distribution | Volume distribution | Regression | 89.048 | 499.265 | 0.04 | 10.00 |
| Excretion | Clearance | Regression | 89.048 | 499.265 | 0.00 | 26.00 |
| Excretion | Half-life of a drug | Classification | 89.048 | 499.265 | N/A | N/A |
| Excretion | Renal excretion (OCT2) | Classification | 89.048 | 499.265 | N/A | N/A |
| General Property | Boiling Point | Regression | 32.026 | 1084.473 | -88.60 | 536.00 |
| General Property | Hydration energy | Regression | 89.048 | 499.265 | -25.47 | 3.43 |
| General Property | logD (octanol-water distribution coefficient) | Regression | 27.011 | 1663.492 | -3.62 | 8.75 |
| General Property | logP (octanol-water partition coefficient) | Regression | 27.011 | 1663.492 | -5.08 | 11.29 |
| General Property | logS (water solubility) | Regression | 27.011 | 1663.492 | -13.17 | 1.70 |
| General Property | logVP (vapor pressure) | Regression | 40.031 | 1084.473 | -13.68 | 5.67 |
| General Property | Melting point | Regression | 27.011 | 1237.920 | -196.00 | 385.00 |
| General Property | pKa | Regression | 32.026 | 1084.473 | -7.10 | 18.08 |
| General Property | pKb | Regression | 32.026 | 1084.473 | -8.00 | 30.90 |
| Metabolism | Breast cancer resistance protein inhibitor (BCRP) | Classification | 89.048 | 499.265 | N/A | N/A |
| Metabolism | Drug metabolism (cyp1a2 inhibitor) | Classification | 27.011 | 1663.492 | N/A | N/A |
| Metabolism | Drug metabolism (cyp1a2 substrate) | Classification | 89.048 | 498.220 | N/A | N/A |
| Metabolism | Drug metabolism (cyp2c19 inhibitor) | Classification | 27.011 | 1663.492 | N/A | N/A |
| Metabolism | Drug metabolism (cyp2c19 substrate) | Classification | 89.048 | 498.220 | N/A | N/A |
| Metabolism | Drug metabolism (cyp2c9 inhibitor) | Classification | 27.011 | 1663.492 | N/A | N/A |
| Metabolism | Drug metabolism (cyp2c9 substrate) | Classification | 89.048 | 499.265 | N/A | N/A |
| Metabolism | Drug metabolism (cyp2d6 inhibitor) | Classification | 27.011 | 1663.492 | N/A | N/A |
| Metabolism | Drug metabolism (cyp2d6 substrate) | Classification | 89.048 | 499.265 | N/A | N/A |
| Metabolism | Drug metabolism (cyp3a4 inhibitor) | Classification | 27.011 | 1663.492 | N/A | N/A |
| Metabolism | Drug metabolism (cyp3a4 substrate) | Classification | 61.992 | 1084.473 | N/A | N/A |
| Metabolism | Hepatic uptake rate (OATP1B1) | Classification | 61.992 | 1084.473 | N/A | N/A |
| Metabolism | Hepatic uptake rate (OATP1B3) | Classification | 73.089 | 707.530 | N/A | N/A |
| Toxicity | Ames | Classification | 27.011 | 1237.920 | N/A | N/A |
| Toxicity | Avian Tox | Classification | 89.048 | 499.265 | N/A | N/A |
| Toxicity | Bee Tox | Classification | 89.048 | 497.204 | N/A | N/A |
| Toxicity | Bioconcentration factor Tox | Regression | 89.048 | 499.265 | -1.70 | 5.69 |
| Toxicity | Biodegradation Tox | Classification | 58.042 | 1084.473 | N/A | N/A |
| Toxicity | Carcinogenicity Tox | Classification | 58.042 | 1084.473 | N/A | N/A |
| Toxicity | Marine Tox (crustacean) | Classification | 89.048 | 499.265 | N/A | N/A |
| Toxicity | Liver Tox (dili) | Classification | 73.089 | 707.530 | N/A | N/A |
| Toxicity | Eye Tox (corrosion) | Classification | 40.031 | 1084.473 | N/A | N/A |
| Toxicity | Eye Tox (irritation) | Classification | 32.026 | 1084.473 | N/A | N/A |
| Toxicity | Maximum recommended daily dose | Regression | 73.089 | 522.083 | -5.00 | 3.00 |
| Toxicity | Marine Tox (Fathead minnow) | Regression | 89.048 | 499.265 | 0.00 | 0.00 |
| Toxicity | Liver Tox (h_ht) | Classification | 40.031 | 1084.473 | N/A | N/A |
| Toxicity | Heart rhythm disorder | Classification | 27.011 | 1663.492 | N/A | N/A |
| Toxicity | Marine Tox (Daphnia magna) | Regression | 89.048 | 498.220 | 0.12 | 10.06 |
| Toxicity | Genetic Tox (micronucleus) | Classification | 89.048 | 499.265 | N/A | N/A |
| Toxicity | NR-AhR Tox | Classification | 30.047 | 1084.473 | N/A | N/A |
| Toxicity | NR-AR Tox | Classification | 27.011 | 1237.920 | N/A | N/A |
| Toxicity | NR-AR-LBD Tox | Classification | 30.047 | 1084.473 | N/A | N/A |
| Toxicity | NR-Aromatase Tox | Classification | 32.026 | 1084.473 | N/A | N/A |
| Toxicity | NR-ER Tox | Classification | 32.026 | 1084.473 | N/A | N/A |
| Toxicity | NR-ER-LBD Tox | Classification | 30.047 | 1237.920 | N/A | N/A |
| Toxicity | NR-GR Tox | Classification | 32.026 | 1084.473 | N/A | N/A |
| Toxicity | NR-PPAR-gamma Tox | Classification | 32.026 | 1084.473 | N/A | N/A |
| Toxicity | NR-TR Tox | Classification | 32.026 | 1084.473 | N/A | N/A |
| Toxicity | 50% Growth inhibition to Tetraphymena pyriformis after 48 hours Tox | Regression | 58.042 | 1084.473 | -2.15 | 6.36 |
| Toxicity | Rat oral acute Tox | Regression | 27.011 | 1663.492 | -0.34 | 7.10 |
| Toxicity | Rat oral chronic Tox | Regression | 89.048 | 498.220 | -2.00 | 3.86 |
| Toxicity | Respiratory Tox | Classification | 32.026 | 1084.473 | N/A | N/A |
| Toxicity | Skin sensitivity | Classification | 89.048 | 499.265 | N/A | N/A |
| Toxicity | SR-ARE Tox | Classification | 32.026 | 1084.473 | N/A | N/A |
| Toxicity | SR-ATAD5 Tox | Classification | 27.011 | 1237.920 | N/A | N/A |
| Toxicity | SR-HSE Tox | Classification | 32.026 | 1084.473 | N/A | N/A |
| Toxicity | SR-MMP Tox | Classification | 32.026 | 1084.473 | N/A | N/A |
| Toxicity | SR-P53 Tox | Classification | 27.011 | 1237.920 | N/A | N/A |

**Table S2. Performance comparison on ADMETlab 2.0 benchmark.** The performance metrics of ADMETlab 2.0 were gathered from the publication(1). Deep-PK's performance was evaluated using 3-fold cross-validation. Improvements were categorised as 'no/weak,' 'reasonable,' and 'significant' if the increase was less than 0.1, less than 0.2 but greater than 0.1, and greater than 0.2, respectively.

| **Name** | **ADMETlab 2.0** | **3-fold CV Deep-PK (average)** | **3-fold CV Deep-PK (std)** | **Metric** | **Category** | **Improvement category** |
| --- | --- | --- | --- | --- | --- | --- |
| Drug metabolism (cyp1a2 substrate) | 0.3 | 0.612 | 0.022 | MCC | Metabolism | significant improvement |
| Drug metabolism (cyp2c19 substrate) | 0.3 | 0.583 | 0.134 | MCC | Metabolism | significant improvement |
| NR-AR Tox | 0.35 | 0.779 | 0.02 | MCC | Toxicity | significant improvement |
| NR-AR-LBD Tox | 0.47 | 0.672 | 0.021 | MCC | Toxicity | significant improvement |
| Ames | 0.61 | 0.719 | 0.007 | MCC | Toxicity | reasonable improvement |
| Drug metabolism (cyp2c9 substrate) | 0.39 | 0.581 | 0.02 | MCC | Metabolism | reasonable improvement |
| Oral bioavailability 20% | 0.41 | 0.59 | 0.03 | MCC | Absorption | reasonable improvement |
| Maximum recommended daily dose | 0.47 | 0.623 | 0.012 | MCC | Toxicity | reasonable improvement |
| Intestinal absorption | 0.69 | 0.811 | 0.041 | MCC | Absorption | reasonable improvement |
| NR-AhR Tox | 0.57 | 0.694 | 0.017 | MCC | Toxicity | reasonable improvement |
| NR-ER Tox | 0.32 | 0.45 | 0.023 | MCC | Toxicity | reasonable improvement |
| NR-ER-LBD Tox | 0.36 | 0.519 | 0.029 | MCC | Toxicity | reasonable improvement |
| NR-PPAR-gamma Tox | 0.34 | 0.462 | 0.093 | MCC | Toxicity | reasonable improvement |
| Drug efflux pump inhibitor (pgp_inhibitor) | 0.72 | 0.837 | 0.012 | MCC | Absorption | reasonable improvement |
| Drug efflux pump substrate (pgp_substrate) | 0.54 | 0.739 | 0.007 | MCC | Absorption | reasonable improvement |
| 50% Growth inhibition to Tetraphymena pyriformis after 48 hours Tox | 0.72 | 0.836 | 0.003 | R2 | Toxicity | reasonable improvement |
| Respiratory Tox | 0.51 | 0.623 | 0.016 | MCC | Toxicity | reasonable improvement |
| Skin sensitivity | 0.46 | 0.587 | 0* | MCC | Toxicity | reasonable improvement |
| SR-ATAD5 Tox | 0.36 | 0.468 | 0.015 | MCC | Toxicity | reasonable improvement |
| SR-HSE Tox | 0.39 | 0.549 | 0.011 | MCC | Toxicity | reasonable improvement |
| SR-P53 Tox | 0.37 | 0.505 | 0.026 | MCC | Toxicity | reasonable improvement |
| BBB (logBB) | 0.72 | 0.737 | 0.006 | MCC | Distribution | no improvement |
| Bioconcentration factor Tox | 0.79 | 0.781 | 0.005 | R2 | Toxicity | no improvement |
| Intestinal permeability (Caco2) logP | 0.75 | 0.787 | 0.005 | MCC | Absorption | no improvement |
| Carcinogenicity Tox | 0.48 | 0.481 | 0.031 | MCC | Toxicity | no improvement |
| Clearance | 0.68 | 0.664 | 0.017 | R2 | Excretion | no improvement |
| Drug metabolism (cyp1a2 inhibitor) | 0.7 | 0.746 | 0.006 | MCC | Metabolism | no improvement |
| Drug metabolism (cyp2c19 inhibitor) | 0.68 | 0.705 | 0.006 | MCC | Metabolism | no improvement |
| Drug metabolism (cyp2c9 inhibitor) | 0.67 | 0.663 | 0.009 | MCC | Metabolism | no improvement |
| Drug metabolism (cyp2d6 inhibitor) | 0.56 | 0.629 | 0.011 | MCC | Metabolism | no improvement |
| Drug metabolism (cyp2d6 substrate) | 0.55 | 0.647 | 0.025 | MCC | Metabolism | no improvement |
| Drug metabolism (cyp3a4 inhibitor) | 0.66 | 0.692 | 0.004 | MCC | Metabolism | no improvement |
| Drug metabolism (cyp3a4 substrate) | 0.44 | 0.444 | 0.012 | MCC | Metabolism | no improvement |
| Liver Tox (dili) | 0.79 | 0.841 | 0* | MCC | Toxicity | no improvement |
| Eye Tox (corrosion) | 0.91 | 0.954 | 0* | MCC | Toxicity | no improvement |
| Eye Tox (irritation) | 0.88 | 0.897 | 0.009 | MCC | Toxicity | no improvement |
| Oral bioavailability 30% | 0.58 | 0.498 | 0.014 | MCC | Absorption | no improvement |
| Marine Tox (Fathead minnow) | 0.75 | 0.767 | 0.002 | R2 | Toxicity | no improvement |
| Fraction unbound in human plasma | 0.76 | 0.719 | 0.005 | R2 | Distribution | no improvement |
| Liver Tox (h_ht) | 0.46 | 0.466 | 0.011 | MCC | Toxicity | no improvement |
| Heart rhythm disorder | 0.78 | 0.786 | 0.008 | MCC | Toxicity | no improvement |
| Marine Tox (Daphnia magna) | 0.52 | 0.565 | 0.018 | R2 | Toxicity | no improvement |
| logD (octanol-water distribution coefficient) | 0.89 | 0.912 | 0.001 | R2 | property | no improvement |
| logP (octanol-water partition coefficient) | 0.96 | 0.965 | 0* | R2 | property | no improvement |
| logS (water solubility) | 0.85 | 0.868 | 0.002 | R2 | property | no improvement |
| Intestinal permeability (MDCK) | 0.73 | 0.773 | 0.006 | R2 | Absorption | no improvement |
| NR-Aromatase Tox | 0.26 | 0.315 | 0.033 | MCC | Toxicity | no improvement |
| Plasma protein binding | 0.73 | 0.765 | 0.008 | R2 | Distribution | no improvement |
| Rat oral acute Tox | 0.55 | 0.579 | 0.003 | MCC | Toxicity | no improvement |
| SR-ARE Tox | 0.47 | 0.552 | 0.01 | MCC | Toxicity | no improvement |
| SR-MMP Tox | 0.66 | 0.735 | 0.017 | MCC | Toxicity | no improvement |
| Half-life of a drug | 0.48 | 0.543 | 0.013 | MCC | Excretion | no improvement |
| Volume distribution | 0.78 | 0.702 | 0.011 | R2 | Distribution | no improvement |

*Endpoint achieved a standard deviation lower than 0.000, resulting in a rounded value of 0.

**Table S3. Performance comparison on toxCSM benchmark.** The performance metrics of toxCSM were collected from the publication(2). Deep-PK's performance was evaluated using 3-fold cross-validation. Improvements were categorised as 'no/weak,' 'reasonable,' and 'significant' if the increase was less than 0.1, less than 0.2 but greater than 0.1, and greater than 0.2, respectively.

| **Name** | **toxCSM** | **3-fold CV Deep-PK (average)** | **3-fold CV Deep-PK (std)** | **Metric** | **Category** | **Improvement category** |
| --- | --- | --- | --- | --- | --- | --- |
| Bee Tox | 0.704 | 0.905 | 0 | MCC | Toxicity | significant improvement |
| Carcinogenicity Tox | 0.666 | 0.893 | 0 | MCC | Toxicity | significant improvement |
| Heart rhythm disorder 1 | 0.566 | 0.818 | 0.0316 | MCC | Toxicity | significant improvement |
| NR-AhR Tox | 0.473 | 0.68 | 0.0257 | MCC | Toxicity | significant improvement |
| NR-Aromatase Tox | 0.295 | 0.538 | 0.0164 | MCC | Toxicity | significant improvement |
| NR-ER Tox | 0.32 | 0.656 | 0.0279 | MCC | Toxicity | significant improvement |
| NR-GR Tox | 0.46 | 0.686 | 0.0199 | MCC | Toxicity | significant improvement |
| Liver Tox (dili) | 0.408 | 0.511 | 0.0164 | MCC | Toxicity | reasonable improvement |
| Marine Tox (Fathead minnow) | 0.716 | 0.883 | 0 | MCC | Toxicity | reasonable improvement |
| Heart rhythm disorder 2 | 0.659 | 0.804 | 0 | MCC | Toxicity | reasonable improvement |
| NR-ER-LBD Tox | 0.527 | 0.627 | 0.0101 | MCC | Toxicity | reasonable improvement |
| NR-TR Tox | 0.316 | 0.444 | 0.0173 | MCC | Toxicity | reasonable improvement |
| Skin sensitivity | 0.652 | 0.797 | 0.026 | MCC | Toxicity | reasonable improvement |
| SR-ARE Tox | 0.392 | 0.565 | 0.0135 | MCC | Toxicity | reasonable improvement |
| SR-ATAD5 Tox | 0.334 | 0.445 | 0.0273 | MCC | Toxicity | reasonable improvement |
| SR-MMP Tox | 0.62 | 0.756 | 0.0205 | MCC | Toxicity | reasonable improvement |
| SR-P53 Tox | 0.405 | 0.577 | 0.0793 | MCC | Toxicity | reasonable improvement |
| Ames | 0.698 | 0.66 | 0.0089 | MCC | Toxicity | no improvement |
| Avian Tox | 0.718 | 0.739 | 0 | MCC | Toxicity | no improvement |
| Biodegradation Tox | 0.73 | 0.787 | 0.0022 | MCC | Toxicity | no improvement |
| Eye Tox (corrosion) | 0.982 | 0.957 | 0.0043 | MCC | Toxicity | no improvement |
| Eye Tox (irritation) | 0.795 | 0.883 | 0 | MCC | Toxicity | no improvement |
| Maximum recommended daily dose | 0.505 | 0.543 | 0.0044 | R2 | Toxicity | no improvement |
| Marine Tox (Fathead minnow) | 0.636 | 0.601 | 0.0058 | R2 | Toxicity | no improvement |
| Liver Tox (h_ht) | 0.582 | 0.643 | 0.0212 | MCC | Toxicity | no improvement |
| Genetic Tox (micronucleus) | 0.843 | 0.918 | 0.0142 | MCC | Toxicity | no improvement |
| NR-AR Tox | 0.571 | 0.636 | 0.0347 | MCC | Toxicity | no improvement |
| NR-AR-LBD Tox | 0.669 | 0.768 | 0.0115 | MCC | Toxicity | no improvement |
| NR-PPAR-gamma Tox | 0.32 | 0.419 | 0.0629 | MCC | Toxicity | no improvement |
| 50% Growth inhibition to Tetraphymena pyriformis after 48 hours Tox | 0.853 | 0.875 | 0.0006 | MCC | Toxicity | no improvement |
| 50% Growth inhibition to Tetraphymena pyriformis after 48 hours Tox | 0.849 | 0.836 | 0.0038 | R2 | Toxicity | no improvement |
| Rat oral acute Tox | 0.617 | 0.646 | 0.0032 | R2 | Toxicity | no improvement |
| Rat oral chronic Tox | 0.646 | 0.711 | 0.0094 | R2 | Toxicity | no improvement |
| SR-HSE Tox | 0.353 | 0.405 | 0.0321 | MCC | Toxicity | no improvement |
| Marine Tox (crustacean) | 0.65 | 0.716 | 0.0391 | MCC | Toxicity | no improvement |
| Respiratory Tox | 0.717 | 0.75 | 0 | MCC | Toxicity | no improvement |

**Table S4. Comparative Performance of Deep-PK and Other Methods Across Multiple Datasets.** The metrics, MCC for classification tasks and R^2^ for regression tasks, were collected from the respective publications for 77 endpoints spanning ADMETlab 2.0(1), Interpretable-ADMET(3), pkCSM(4), and toxCSM(2). Deep-PK's performance was evaluated using a 3-fold cross-validation and results were averaged.  'N/A' indicates if there are no predictive models available for comparison.

| **Category** | **Name** | **3-fold CV Deep-PK (average)** | **3-fold CV Deep-PK (std)** | **ADMETlab2.0** | **Interpretable-ADMET** | **toxCSM** | **Metric** |
| --- | --- | --- | --- | --- | --- | --- | --- |
| Absorption | Intestinal permeability (Caco2) logP | 0.76 | 0.009 | N/A | N/A | N/A | R2 |
| Absorption | Oral bioavailability 20% | 0.47 | 0.003 | 0.41 | N/A | N/A | MCC |
| Absorption | Intestinal permeability (MDCK) | 0.71 | 0.005 | 0.731 | N/A | N/A | R2 |
| Absorption | Oral bioavailability 50% | 0.45 | 0.008 | N/A | 0.342 | N/A | MCC |
| Absorption | Drug efflux pump inhibitor (pgp_inhibitor) | 0.76 | 0.016 | 0.72 | 0.749 | N/A | MCC |
| Absorption | Drug efflux pump substrate (pgp_substrate) | 0.79 | 0 | 0.54 | 0.57 | N/A | MCC |
| Absorption | Drug efflux pump inhibitor (pgp_inhibitor1) | 0.76 | 0.022 | N/A | N/A | N/A | MCC |
| Absorption | Drug efflux pump inhibitor (pgp_inhibitor2) | 0.87 | 0.015 | N/A | N/A | N/A | MCC |
| Absorption | Intestinal absorption | 0.93 | 0 | 0.69 | 0.9 | N/A | MCC |
| Absorption | Skin permeability | 0.37 | 0 | N/A | N/A | N/A | R2 |
| Distribution | BBB (CNS) | 0.67 | 0.015 | N/A | N/A | N/A | R2 |
| Distribution | BBB (logBB) | 0.95 | 0.011 | 0.72 | 0.771 | N/A | MCC |
| Distribution | Fraction unbound in human plasma | 0.59 | 0.012 | 0.763 | N/A | N/A | R2 |
| Distribution | Plasma protein binding | 0.55 | 0 | 0.733 | 0.563 | N/A | R2 |
| Distribution | Volume distribution | 0.78 | 0.003 | 0.782 | N/A | N/A | R2 |
| Excretion | Clearance | 0.76 | 0.009 | 0.678 | N/A | N/A | R2 |
| Excretion | Renal excretion (OCT2) | 0.78 | 0.025 | N/A | 0.347 | N/A | MCC |
| Excretion | Half-life of a drug | 0.32 | 0.056 | 0.48 | 0.358 | N/A | MCC |
| General Property | Boiling Point | 0.97 | 0.001 | N/A | 0.886 | N/A | R2 |
| General Property | Hydration energy | 0.97 | 0 | N/A | 0.923 | N/A | R2 |
| General Property | logD (octanol-water distribution coefficient) | 0.91 | 0.002 | 0.892 | 0.777 | N/A | R2 |
| General Property | logP (octanol-water partition coefficient) | 0.96 | 0.001 | 0.957 | 0.928 | N/A | R2 |
| General Property | logS (water solubility) | 0.82 | 0.002 | 0.854 | 0.785 | N/A | R2 |
| General Property | logVP (vapor pressure) | 0.95 | 0.002 | N/A | 0.863 | N/A | R2 |
| General Property | Melting point | 0.86 | 0.001 | N/A | 0.795 | N/A | R2 |
| General Property | pKa | 0.78 | 0.008 | N/A | 0.722 | N/A | R2 |
| General Property | pKb | 0.82 | 0.008 | N/A | 0.676 | N/A | R2 |
| Metabolism | Breast cancer resistance protein inhibitor (BCRP) | 0.44 | 0 | N/A | 0.491 | N/A | MCC |
| Metabolism | Drug metabolism (cyp1a2 inhibitor) | 0.67 | 0.008 | 0.7 | 0.585 | N/A | MCC |
| Metabolism | Drug metabolism (cyp1a2 substrate) | 0.69 | 0.054 | 0.3 | N/A | N/A | MCC |
| Metabolism | Drug metabolism (cyp2c19 inhibitor) | 0.65 | 0.011 | 0.68 | 0.533 | N/A | MCC |
| Metabolism | Drug metabolism (cyp2c19 substrate) | 0.65 | 0.028 | 0.3 | N/A | N/A | MCC |
| Metabolism | Drug metabolism (cyp2c9 inhibitor) | 0.6 | 0.013 | 0.67 | 0.504 | N/A | MCC |
| Metabolism | Drug metabolism (cyp2c9 substrate) | 0.43 | 0.033 | 0.39 | 0.176 | N/A | MCC |
| Metabolism | Drug metabolism (cyp2d6 inhibitor) | 0.6 | 0.012 | 0.56 | 0.474 | N/A | MCC |
| Metabolism | Drug metabolism (cyp2d6 substrate) | 0.48 | 0.044 | 0.55 | 0.34 | N/A | MCC |
| Metabolism | Drug metabolism (cyp3a4 inhibitor) | 0.6 | 0.009 | 0.66 | 0.525 | N/A | MCC |
| Metabolism | Drug metabolism (cyp3a4 substrate) | 0.64 | 0.009 | 0.44 | 0.228 | N/A | MCC |
| Metabolism | Hepatic uptake rate (OATP1B1) | 0.59 | 0.011 | N/A | 0.434 | N/A | MCC |
| Metabolism | Hepatic uptake rate (OATP1B3) | 0.54 | 0.02 | N/A | 0.425 | N/A | MCC |
| Toxicity | Ames | 0.73 | 0.002 | 0.61 | 0.631 | 0.698 | MCC |
| Toxicity | Avian Tox | 0.88 | 0.034 | N/A | N/A | 0.718 | MCC |
| Toxicity | Bee Tox | 0.8 | 0 | N/A | 0.287 | 0.704 | MCC |
| Toxicity | Bioconcentration factor Tox | 0.89 | 0.002 | 0.786 | N/A | N/A | R2 |
| Toxicity | Biodegradation Tox | 0.76 | 0.015 | N/A | 0.652 | 0.73 | MCC |
| Toxicity | Carcinogenicity Tox | 0.52 | 0.024 | 0.48 | 0.321 | 0.666 | MCC |
| Toxicity | Marine Tox (crustacean) | 0.65 | 0.013 | N/A | N/A | N/A | MCC |
| Toxicity | Liver Tox (dili) | 0.37 | 0.014 | 0.79 | 0.307 | 0.408 | MCC |
| Toxicity | Eye Tox (corrosion) | 0.94 | 0.004 | 0.91 | 0.904 | 0.982 | MCC |
| Toxicity | Eye Tox (irritation) | 0.9 | 0.007 | 0.88 | 0.835 | 0.795 | MCC |
| Toxicity | Maximum recommended daily dose | 0.57 | 0.013 | N/A | N/A | 0.505 | R2 |
| Toxicity | Marine Tox (Fathead minnow) | 0.16 | 0.017 | 0.745 | N/A | 0.636 | R2 |
| Toxicity | Liver Tox (h_ht) | 0.37 | 0.006 | 0.46 | 0.246 | 0.582 | MCC |
| Toxicity | Heart rhythm disorder | 0.74 | 0.002 | 0.78 | 0.545 | N/A | MCC |
| Toxicity | Heart rhythm disorder1 | 0.68 | 0.101 | N/A | N/A | 0.566 | MCC |
| Toxicity | Heart rhythm disorder2 | 0.68 | 0.022 | N/A | N/A | 0.659 | MCC |
| Toxicity | Marine Tox (Daphnia magna) | 0.68 | 0.004 | 0.524 | N/A | N/A | R2 |
| Toxicity | Genetic Tox (micronucleus) | 0.8 | 0.05 | N/A | N/A | 0.843 | MCC |
| Toxicity | NR-AhR Tox | 0.59 | 0.007 | 0.57 | 0.517 | 0.473 | MCC |
| Toxicity | NR-AR Tox | 0.73 | 0.012 | 0.35 | N/A | 0.571 | MCC |
| Toxicity | NR-AR-LBD Tox | 0.87 | 0 | 0.47 | 0.705 | 0.669 | MCC |
| Toxicity | NR-Aromatase Tox | 0.51 | 0.021 | 0.26 | 0.42 | 0.295 | MCC |
| Toxicity | NR-ER Tox | 0.5 | 0.008 | 0.32 | N/A | 0.32 | MCC |
| Toxicity | NR-ER-LBD Tox | 0.64 | 0.034 | 0.36 | 0.39 | 0.527 | MCC |
| Toxicity | NR-GR Tox | 0.56 | 0.022 | N/A | N/A | 0.46 | MCC |
| Toxicity | NR-PPAR-gamma Tox | 0.31 | 0.02 | 0.34 | 0.245 | 0.32 | MCC |
| Toxicity | NR-TR Tox | 0.46 | 0.036 | N/A | N/A | 0.316 | MCC |
| Toxicity | 50% Growth inhibition to Tetraphymena pyriformis after 48 hours Tox | 0.86 | 0.002 | 0.723 | 0.832 | 0.849 | R2 |
| Toxicity | Rat oral acute Tox | 0.67 | 0.005 | N/A | N/A | 0.617 | R2 |
| Toxicity | Rat oral chronic Tox | 0.55 | 0.031 | N/A | N/A | 0.646 | R2 |
| Toxicity | Respiratory Tox | 0.6 | 0.022 | 0.51 | N/A | N/A | MCC |
| Toxicity | Skin sensitivity | 0.56 | 0.028 | 0.46 | 0.623 | 0.652 | MCC |
| Toxicity | SR-ARE Tox | 0.53 | 0.02 | 0.47 | 0.41 | 0.392 | MCC |
| Toxicity | SR-ATAD5 Tox | 0.57 | 0.01 | 0.36 | 0.281 | 0.334 | MCC |
| Toxicity | SR-HSE Tox | 0.39 | 0.025 | 0.39 | 0.414 | 0.353 | MCC |
| Toxicity | SR-MMP Tox | 0.7 | 0.009 | 0.66 | 0.58 | 0.62 | MCC |
| Toxicity | SR-P53 Tox | 0.5 | 0.01 | 0.37 | 0.366 | 0.405 | MCC |

**REFERENCES**

1. Xiong, G., Wu, Z., Yi, J., Fu, L., Yang, Z., Hsieh, C., Yin, M., Zeng, X., Wu, C., Lu, A. *et al.* (2021) ADMETlab 2.0: an integrated online platform for accurate and comprehensive predictions of ADMET properties. *Nucleic Acids Res*, **49**, W5-w14.

2. Sá, A.G.C.d., Long, Y., Portelli, S., Pires, D.E.V. and Ascher, D.B. (2022) toxCSM: comprehensive prediction of small molecule toxicity profiles. *Brief. Bioinform.*, **23**, bbac337.

3. Wei, Y., Li, S., Li, Z., Wan, Z. and Lin, J. (2022) Interpretable-ADMET: a web service for ADMET prediction and optimization based on deep neural representation. *Bioinformatics*, **38**, 2863-2871.

4. Pires, D.E., Blundell, T.L. and Ascher, D.B. (2015) pkCSM: Predicting Small-Molecule Pharmacokinetic and Toxicity Properties Using Graph-Based Signatures. *J Med Chem*, **58**, 4066-4072.

5. Daina, A., Michielin, O. and Zoete, V. (2017) SwissADME: a free web tool to evaluate pharmacokinetics, drug-likeness and medicinal chemistry friendliness of small molecules. *Sci Rep*, **7**, 42717.
